# Piquing Curiosity: Déjà vu-Like States Are Associated with Feelings of Curiosity and Information-Seeking Behaviors

**DOI:** 10.3390/jintelligence11060112

**Published:** 2023-06-05

**Authors:** Katherine L. McNeely-White, Anne M. Cleary

**Affiliations:** 1 Department of Neurology, University of California, Davis, CA 95618, USA; 2 Department of Psychology, Colorado State University, Fort Collins, CO 80523, USA; anne.cleary@colostate.edu

**Keywords:** curiosity, metacognition, familiarity-detection, déjà vu, retrieval failure, recognition memory

## Abstract

Curiosity during learning increases information-seeking behaviors and subsequent memory retrieval success, yet the mechanisms that drive curiosity and its accompanying information-seeking behaviors remain elusive. Hints throughout the literature suggest that curiosity may result from a metacognitive signal—possibly of closeness to a not yet accessible piece of information—that in turn leads the experiencer to seek out additional information that will resolve a perceptibly small knowledge gap. We examined whether metacognition sensations thought to signal the likely presence of an as yet unretrieved relevant memory (such as familiarity or déjà vu) might be involved. Across two experiments, when cued recall failed, participants gave higher curiosity ratings during reported déjà vu (Experiment 1) or déjà entendu (Experiment 2), and these states were associated with increased expenditure of limited experimental resources to discover the answer. Participants also spent more time attempting to retrieve information and generated more incorrect information when experiencing these déjà vu-like states than when not. We propose that metacognition signaling of the possible presence of an as yet unretrieved but relevant memory may drive curiosity and prompt information-seeking that includes further search efforts.

## 1. Introduction

Feelings of curiosity are foundational to learning and memory, with curiosity potentially motivating information-seeking behaviors; in turn, self-motivated information-seeking subsequently strengthens the encoding of the to-be-learned information (e.g., Gruber et al. 2014; Kang et al. 2009; Wade and Kidd 2019). Research has demonstrated that information that originally prompted intense levels of curiosity is more likely to be recollected in a subsequent memory test even after a delay of two weeks (Kang et al. 2009). Further, Gruber et al. showed that, when experiencing intense levels of curiosity, participants were more likely to encode incidental information, such as a face interleaved between to-be-learned trivia information (see also Murphy et al. 2021). Findings such as these have implications for learning in educational settings, including informing instructors on how best to motivate learners within classroom settings (e.g., Arnone and Small 1995; Engel 2011, 2015; Lindholm 2018; Malone 1981; Maw and Maw 1966; Pluck and Johnson 2011).

The mechanisms and origins of curiosity, though, are still not well understood. Gruber and Ranganath (2019) argue that curiosity is a cognitive state, and some theorists have proposed that curiosity is a momentary emotional–motivational state that emerges due to metacognitive monitoring (Litman 2009), which is awareness of one’s own cognitive processes (e.g., Koriat 2007; Nelson and Narens 1990). Specifically, Litman (2009) proposed that curiosity arises from metacognitive monitoring, emerging due to a perceived gap between one’s current knowledge needs and the current accessible knowledge state; in turn, this perceived gap motivates the person to resolve the gap, with curiosity serving as an emotional–motivational experience that is directly influenced by one’s metacognitive processes. In this view, curiosity is thought to involve a match between the to-be-learned information and the participant’s capacity or urgent need to encode or discover it (e.g., Wade and Kidd 2019). Importantly, curiosity does not appear to result from merely realizing that one does not know something. Research suggests that curiosity increases as one feels closer to attaining the as yet unknown piece of information. For example, curiosity has been shown to increase with (1) having pieces of relevant information available (Van Dijk and Zeelenberg 2007), (2) having high relevant knowledge related to the topic at hand (Witherby and Carpenter 2022), and (3) feeling as if a piece of information is on the tip of one’s tongue (Metcalfe et al. 2017). These types of findings have led to the supposition that curiosity can result from a feeling-of-closeness to the as yet unattained information (Litman et al. 2005; Loewenstein 1994; Metcalfe et al. 2020; Noordewier and van Dijk 2020; Van Dijk and Zeelenberg 2007). From this perspective, if one feels close to the unattained information, the likelihood of curiosity should be high.

An unexplored possible source of curiosity that fits with this perspective is déjà vu—the feeling of having experienced something before despite that simultaneously seeming impossible (see Cleary and Brown 2022, for a review). Although déjà vu is a seemingly quirky aspect of memory, Schwartz and Cleary (2016) argued that a possible adaptive purpose of feelings of déjà vu is to prompt a search for relevant information in memory that could indicate why the situation seems so familiar. This idea, taken together with the theory that curiosity involves a feeling-of-closeness to an unattained piece of information, led us to investigate the possibility that déjà vu (and déjà vu-like states) involve curiosity. Toward this end, in the present study, we examined whether feelings of déjà vu (and its auditory analog—déjà entendu) are associated with curiosity and information-seeking behaviors.

### 1.1. Metacognition

Metacognition is the ability to think about one’s own cognitive processes, knowledge, and memory processes (e.g., Koriat 2007; Nelson and Narens 1990; Rhodes 2019). It is thought to consist of two components: monitoring and control (Nelson and Narens 1990). *Monitoring* involves the act of reflecting on one’s current state of knowledge or cognitive processes, while *control* involves decisions made about how to proceed in light of the information gleaned from the monitoring. Gruber and Ranganath’s (2019) suggestion that curiosity is a cognitive state and Litman’s (2009) proposal that curiosity emerges due to metacognitive processes both fit with the idea that curiosity may be the result of metacognitive monitoring. Specifically, the state of curiosity may arise from inner reflection on one’s own knowledge in relation to a current problem at hand (the monitoring component of metacognition). In turn, the emergence of the state of curiosity might motivate a desire to discover the as yet unknown piece of information and expend resources to engage in information-seeking behaviors (the control aspect of metacognition). In the present study, we aim to arrive at a better understanding of the metacognitive monitoring factors that can lead to the state of curiosity and its corresponding information-seeking behaviors. In our effort to do so, we build upon a relatively recent theoretical suggestion in the literature—that curiosity may be driven by metacognitive feelings of being close to accessing a piece of information that has not yet been forthcoming (Litman 2019; Metcalfe et al. 2020, 2022).

### 1.2. Theories of Curiosity

Until quite recently, curiosity and metacognition studies have been largely separate from one another (see Goupil and Proust 2023 for a comprehensive review connecting the two domains). However, as with many cognitive phenomena, curiosity has been a topic of study since the dawn of cognitive psychology, with researchers examining not just the circumstances under which it occurs and the consequences of it, but also why humans exhibit curiosity and information-seeking behavior (e.g., Berlyne 1950, 1958, 1960, 1962, 1966).

Curiosity has been described as a desire to know or experience new information, whereby accessing that information results in a reward, whether it be an external reward, such as discovering a new piece of information that will ensure survival, or an internal reward, such as the resolution of uncertainty or merely the pleasure found in acquiring information (FitzGibbon et al. 2020; Kang et al. 2009; Litman 2005). In fact, the notion of merely finding pleasure in acquiring non-essential information is what has puzzled researchers of curiosity for some time, as it seems at odds with the more evolutionarily plausible purpose of curiosity: to explore one’s environment and discover crucial pieces of information for survival (e.g., Litman 2005; Silvia 2012). For this reason, a number of theories have attempted to capture the purpose, function, and phenomenology of curiosity as a cognitive construct.

#### 1.2.1. Curiosity Drive Theory

One of the earliest classes of theories examining curiosity is known as *curiosity-drive theory*, sometimes referred to as *drive reduction theory* (see Litman 2005). This class of theories proposes that curiosity can be equated with rather unpleasant experiences of uncertainty and reducing those feelings of uncertainty is rewarding. The main assumption behind this class of theories is that humans strive for coherence, such that there are little to no unknowns in their environment, as those could be potentially threatening for survival. Thus, whenever one encounters a novel, complex, and/or ambiguous stimulus, such as a new peculiar insect, this elicits a sense of uncomfortable uncertainty that must be resolved. The individual will seek out information, such as by inspecting or interacting with the stimulus, in order to learn about its properties and characteristics, leading to the resolution of uncertainty that could potentially threaten survival. Indeed, early research on curiosity provided support for this viewpoint, as can be seen in a series of experiments summarized by Berlyne (1966), who described curiosity as a “condition of discomfort, due to inadequacy of information, that motivates specific exploration” (p. 26).

In his experiments examining curiosity and information-seeking behaviors for visual stimuli, Berlyne (1958) presented participants with images of varying levels of ambiguity. The images he used were of animals, such as an elephant or bird, but were altered in a way, such that some of the animal’s features, such as the legs, were incongruent. Participants were presented with two images side-by-side, with one being the image of a congruent animal (e.g., a tiger) and the other being the altered, incongruent version of that animal (e.g., a tiger with the body of a camel). In monitoring eye movements and fixations, Berlyne found that participants tended to spend more time fixating on the ambiguous, strange animal containing incongruent body parts.

From these patterns of results, Berlyne (1966) proposed that, when presented with surprising, complex, or ambiguous stimuli, people will experience perceptual curiosity, which is interpreted as subjective uncertainty. As this is an aversive state, resolving any feelings of curiosity is desirable. Beyond perceptual curiosity, though, Berlyne also proposed that individuals can experience conceptual conflicts that lead to curiosity-resolving behaviors, such as when internal thought processes conflict, which he coined “epistemic curiosity.” This form of curiosity is thought to reflect a more intense desire to acquire new information, and this thus motivates exploratory behaviors that result in knowledge acquisition (Litman 2005). Of specific interest to the current study is the finding that participants tended to be most curious about information that they felt was most familiar to them (Berlyne 1954).

In his 1954 study examining epistemic curiosity, Berlyne first presented participants with a list of questions concerning invertebrate animals with the task being that they were to choose the correct answer from two potential answers. Participants were also prompted to indicate to which questions they would most prefer to know the answer. Upon completing this list of questions, participants reviewed a list of statements about invertebrate animals, which contained the correct answers to the previously presented list. Finally, participants were given the first list of questions again, but in the form of a free recall task. Berlyne’s results showed that participants were more likely to correctly produce the answer in the final free recall phase of the experiment for questions that prompted a desire to learn the answer (interpreted as curiosity) during the initial forced-choice recognition phase, suggesting that curiosity serves as an adaptive cognitive function for future memory access. Additionally, of great interest to the current study was Berlyne’s finding that, during the initial forced-choice recognition phase, participants were most curious about questions for which they indicated felt most familiar. When participants felt a sense of familiarity with the concept, this led to the greatest level of conflict, as it signaled that they were missing information for something that was stored within memory. Collectively, these results provide an early link between familiarity-detection, a form of metacognition, and feelings of curiosity.

Berlyne (1950) proposed that not all novel stimuli prompt a sense of curiosity, but rather the ones that contain familiar *and* novel elements are the ones most likely to elicit curiosity. Berlyne (1960) proposed that instances of *relative novelty*, in which a novel stimulus contains familiar patterns of elements, might prompt greater feelings of curiosity, as it signals conflict to the individual that their knowledge structures are not complete in terms of the current concept. This might encourage further information-seeking behaviors in order to resolve the conflict and learn new, important information that might fill the gap in terms of what is missing.

While curiosity-drive theories offered a compelling explanation of the function of curiosity, specifically that it motivates the individual to engage in information-seeking behaviors, there is extant research that offers a challenge to curiosity-drive theories. Specifically, a common, universally observed behavior in both non-human animals and humans is that organisms tend to seek out new or novel situations (Litman 2005). Whenever an individual is in an environment that lacks novel or complex stimuli, they are motivated to seek out stimuli that offer new, ambiguous, or complex information (i.e., uncertainty). This frequently observed behavior is a challenge for curiosity-drive theorists, whose central tenet is that individuals seek to reduce uncertainty, and thus resolve curiosity. If individuals are only engaging in information-seeking behaviors to find certainty, then why would they also engage in information-seeking behaviors to find situations that present uncertainty?

#### 1.2.2. Optimal-Arousal Theory

The shortcomings of curiosity-drive theories inspired an alternative account to why organisms demonstrate information-seeking behavior even when the information is not relevant for survival and there are currently no elements prompting a sense of uncertainty. These *optimal-arousal theories* proposed that organisms aim to have an optimal level of arousal in which they are not over- or under-aroused, as both situations are unpleasant (Litman 2005; Litman and Jimerson 2004; Silvia 2012). The optimal-arousal theories are similar to curiosity-drive theories in that they encompass the behaviors that organisms exhibit when faced with uncertainty (e.g., a highly ambiguous stimulus that results in over-stimulation), but with the added component of boredom. When individuals lack arousal and feel bored, they tend to seek out new experiences or stimuli that generate arousal and positive feelings of curiosity. As opposed to the curiosity-drive theorists, optimal-arousal theorists propose that the experience of curiosity itself is rewarding and involves feelings of interest and pleasure rather than negative feelings of uncertainty, although those can still occur. Individuals are motivated to seek out and engage in the learning of new information, as this is considered to be the optimal level of arousal.

Although the optimal-arousal theories are not without their own shortcomings (see Silvia 2012 for a detailed discussion), a major contribution of the optimal-arousal theories is its proposal that curiosity can emerge from boredom, not just due to survival-threatening uncertainty, which inspired a new way of thinking about curiosity. Specifically, it suggested that curiosity can occur due to both a drive-reduction state and an induction (or optimal arousal) state (Litman 2005). This new perspective on curiosity, as discussed below, offers a means by which to frame curiosity as a form of metacognition, which is a primary goal of the current study.

#### 1.2.3. Information Gap Theory

Malone (1981) proposed that humans can experience both sensory curiosity (due to surprising or complex visual patterns in the world) and cognitive curiosity (the prospect of modifying higher-order cognitive knowledge structures). Malone argued that one of the main purposes of curiosity is to signal opportunity to the individual. In other words, curiosity signals to the experiencer that their current knowledge structure lacks completeness or contains a gap, but that there is an opportunity to discover and learn new information that can be used to fill this gap. Along similar lines, Loewenstein (1994) proposed that people might experience curiosity due to metacognitive monitoring. When people detect that there is an information gap (formally called *information gap theory*), they seek to fill that void (exerting metacognitive control). The size of the information or knowledge gap is inversely related to the intensity of curiosity. In short, the smaller the perceived gap (or the closer one feels to arriving at the needed information), the greater the curiosity. Information that is perceived to be very far from the current state of knowledge results in low levels of curiosity because the learner senses having only an incredibly small amount of knowledge about the topic and thus feels overwhelmed about the amount of information that must be learned in order to fill the void. It is when a person feels as if they are on the verge of discovering, learning, or accessing the needed information that high or intense feelings of curiosity occur. In turn, the strong sense of curiosity prompts the person to search further until resolution is found.

#### 1.2.4. Conflict Detection

Gruber and Ranganath (2019) recently proposed a theory that could be considered a variant of Information Gap Theory. Specifically, they proposed the Prediction Appraisal Curiosity Exploration (PACE) framework, according to which the initial trigger of curiosity is a prediction error, whereby a person makes an error in their anticipation of something, and the conflict detection that occurs in response to the incongruency between the anticipated outcome and the actual outcome leads the experiencer to seek resolution through further information-seeking and exploration. According to this view, curiosity (and its corresponding information-seeking) is primarily brought on by conflict detection (also see Gruber and Fandakova 2021).

#### 1.2.5. Interest-Deprivation Theory

Building on the aforementioned theories of curiosity, such as curiosity drive theories (e.g., Berlyne 1950, 1958, 1960, 1962, 1966), optimal arousal theories (e.g., Litman 2005; Litman and Jimerson 2004; Silvia 2012), and Loewenstein’s (1994) information gap theory, Litman and Jimerson (2004) developed the *interest-deprivation theory of curiosity*. They propose that curiosity can emerge due to either the individual feeling as though they are deprived of information and wish to reduce or resolve that gap, or due to feeling a general interest that is not caused by a specific deficit or threat, but rather an enjoyment of learning something new. The latter form of curiosity, called *curiosity as a feeling-of-interest*, involves positive feelings of interest and joy, along with the anticipation of learning something new. Litman and Jimerson proposed that curiosity as a feeling-of-interest is experienced when the person does not feel as though they are suffering from a lack of knowledge, but rather that it would be pleasurable to discover something new and avoid boredom. In a similar vein as the drive-reduction theory and optimal-arousal theory, curiosity as a feeling-of-interest is associated with the anticipated pleasure of finding out new information.

#### 1.2.6. Curiosity as a Feeling-of-Deprivation

Of primary interest to the current study, though, is the form of curiosity that Litman and Jimerson (2004) coined curiosity *as a feeling-of-deprivation*, during which the individual senses a lack of knowledge. They proposed that curiosity as a feeling-of-deprivation is a much more intense curiosity experience that involves the individual sensing that the needed information is substantive, meaningful, and could increase their subjective feelings of competence. This might involve learning the answer to a complex question, a valuable fact, or perhaps the solution to a difficult problem. Crucially, they hypothesize that curiosity as a feeling-of-deprivation is related to very intense feelings of curiosity that more strongly motivate information-seeking behaviors. As the individual perceives themselves as being closer to obtaining the piece of missing information, they experience more intense levels of curiosity, as the needed information is on the verge of being accessed (Noordewier and van Dijk 2020). 

As previously mentioned, Berlyne (1950, 1960) proposed that curiosity may result from a conflict between familiar and novel elements of a stimulus, prompting the individual to engage in information-seeking behaviors in order to resolve the uncertainty. By incorporating the information-gap theory, this proposal by Berlyne is plausible, as the individual may sense that, despite the current stimulus having familiar components, there are unknown aspects about it, signaling to the individual that they have a gap in knowledge, while, at the same time, the familiar components signal to the individual that the gap may be small; that is, the person may be on the verge of accessing the needed information to fill the gap. As discussed by Litman (2019), the self-awareness that one possesses a knowledge gap (and assessing the size of that gap) is a type of metacognitive judgement, as it requires that one reflects on their current level of knowledge and then engage in search processes, either internal or external, in order to fill that gap and learn more about the novel elements of the current stimulus that also somehow feels familiar.

#### 1.2.7. Region of Proximal Learning Framework of Curiosity

Loewenstein’s (1994) information gap theory specifically proposed that one’s perceived proximity to the needed knowledge is what determines the intensity of the curiosity experience. Specifically, people may feel the most curious about information that is not currently accessible but feels as if it is *almost* accessible. As the perceived gap in knowledge narrows, the person feels extremely curious, as the information is close and almost retrievable, and therefore the person feels motivated to discover the needed information (see also Noordewier and van Dijk 2020). In consideration of this, it may be that any metacognitive sensations signaling to the individual that they are close to accessing the information are a necessary component for triggering feelings of curiosity, henceforth referred to as *curiosity as a feeling-of-closeness*, as information that does not feel close to being accessed will not signal to the individual that it is within the realm of possibility to discover, and they will therefore not feel curious to discover the missing information. It is this approach to curiosity that motivated the present study.

A recently proposed framework that integrates the components of prior curiosity theories, while also building on the idea that curiosity emerges when the experiencer feels on the verge of accessing relevant information, is the Region of Proximal Learning Framework of Curiosity (Metcalfe 2023; Metcalfe et al. 2017, 2020, 2022). The central feature of the region of proximal learning framework is that people are the most curious when they feel that they are close to knowing the desired target information. In this framework, in order to arrive at a decision (such as deciding on the answer to a question), an initial probe leads the experiencer to compile all available information, both internal and external, that is relevant to the probe. If the compiled information is sufficient enough to produce an answer, then the experiencer does so and does not have any changes in curiosity. However, if they are unable to produce an answer, or receive feedback that their provided answer was wrong, then they must make a metacognitive judgment as to their next action – give up and mind wander, which occurs when the perceived gap in knowledge is sensed to be quite large, or engage in information-seeking behaviors to resolve the gap, which occurs when the experiencer senses that the compiled information is *almost* sufficient. In these moments, when the experiencer is in their region of proximal learning, they will experience heightened levels of curiosity, which motivate them to seek out and learn further information, potentially resulting in the reward of achieving the desired knowledge state. According to this framework, people will experience the strongest levels of curiosity when their metacognitive experiences suggest that they *almost* know the answer. In other words, they have a sense of curiosity as a result of a metacognitive feeling-of-closeness to the sought after information.

In a compelling series of experiments examining curiosity and confidence, Metcalfe et al. (2022) presented participants with general knowledge questions that were each accompanied by prompts concerning whether the participant knew the answer, how curious they were, and how confident they were about their answer. When examining the relationship between confidence and curiosity, Metcalfe et al. found, unsurprisingly, that when the participant was indeed correct in their answer, curiosity and confidence followed an inverted-U relationship, such that the highest levels of confidence (e.g., 90% confident) were accompanied by lower levels of curiosity when the knowledge gap had been bridged. In short, when the participant did indeed know the answer (without receiving feedback), they were not very curious on trials associated with high confidence. However, the fascinating results are those concerning the curiosity and confidence ratings when the participant was wrong in their response. Unlike the inverted-U relationship that curiosity and confidence followed when the participant was correct, the relationship between curiosity and confidence when the participant was wrong (i.e., the knowledge gap had not yet been resolved) did not follow the same inverted-U relationship as when the participant was correct. At high levels of confidence (e.g., 90% confident), participants provided higher curiosity ratings during trials on which they were wrong in their answer as opposed to when they were correct. Critically, this relationship emerged prior to receiving feedback. The participants had not received feedback concerning whether or not their provided response was accurate, yet there was a difference in the provided curiosity ratings, as if there was some level of unconscious knowledge that their knowledge gap was not resolved, with feelings of curiosity signaling this discrepancy. In other words, it was as if they could detect that they were close to achieving the target knowledge state, but were not quite there yet.

#### 1.2.8. What Elicits a Feeling of Closeness?

A current gap in the literature concerns how it is that people are able to metacognitively detect that they are close to arriving at a piece of information that will resolve a current gap in knowledge. The notion that there may be something akin to a feeling of closeness to a desired piece of as yet inaccessible information is reminiscent of the metacognitive phenomenon known as the tip-of-the-tongue (TOT) state.

### 1.3. Tip-of-the-Tongue (TOT) States

A TOT state occurs when a person feels on the verge of retrieving a word that currently eludes them; the word is said to feel “on the tip of the tongue,” as if about to come to mind at any moment (e.g., Brown 2012; Schwartz 2002a). Indeed, recent research has suggested that the TOT state involves a feeling of closeness to the sought after target information (Rousseau and Kashur 2021), and a growing body of studies suggests that TOT states are linked to increased curiosity to discover the sought after information relative to when there is no TOT state for an unknown answer (Metcalfe et al. 2017) and to corresponding information-seeking behaviors (Cleary et al. 2021b; Metcalfe et al. 2017; Schwartz 2002b).

#### 1.3.1. An Association between TOT States and Curiosity

A study conducted by Litman et al. (2005) provided initial evidence for a relationship between feelings of curiosity and TOT states. In their experiment, participants were presented with general knowledge questions and asked to make a “Know”, “TOT”, or “Don’t Know” response, along with the intensity of their confidence in being able to select the correct answer from a list of potential options and how they were curious to see the correct answer. Upon completing the general knowledge phase (and potentially a recognition memory test), all participants completed an exploratory behavior phase, during which they were given envelopes containing all of the correct answers to the general knowledge questions. Participants were told that they were free to open any of the envelopes but should focus on opening the ones for which they were genuinely interested in seeing the answers. Of most interest to the current study was the finding that participants were more curious to discover the unretrievable answer to a general knowledge question and were subsequently more willing to use limited resources to discover that answer when experiencing a TOT for that answer than when not. Additionally, when participants made a “Don’t Know” response, they showed intermediate levels of curiosity and information-seeking behaviors. “I Know” responses were accompanied by the lowest levels of curiosity and information-seeking behaviors. However, Litman et al.’s design choices did not allow for a clear connection between in-the-moment decision-making processes and TOT states, as participants were given the opportunity to discover the answers at the end of the experiment as opposed to in the moment of retrieval failure. Additionally, Litman et al. only presented 12 general knowledge questions in their experiment.

In a follow-up to this initial study, Metcalfe et al. (2017) used a larger number of stimuli (82 general knowledge questions) and a design that allowed for the examination of in-the-moment changes to decision-making processes as a result of TOT states. After attempting to answer each question, participants were asked to indicate whether they were in a TOT state and also whether they were curious to discover the answer. However, participants were informed that, although they could see the correct answer at a later time, they could only see the correct answer for up to 10% of the questions. Overall, although this design did not allow for immediate resolution to participants’ perceived knowledge gaps, Metcalfe et al. found that, when failing to recall the correct answer, participants were twice as likely to want to see the answer when they were in a TOT state as opposed to a non-TOT state, suggesting that participants are more curious when experiencing a TOT state and are more inclined to devote limited experimental resources toward discovering the target.

#### 1.3.2. An Association between TOT States and Information-Seeking Behavior

The idea that TOTs might prompt information-seeking behavior has been supported in other studies as well. Schwartz (2002b) found that participants were more likely to engage in retrieval search efforts when experiencing a TOT relative to when not. Additionally, Cleary et al. (2021b) found that, when offered the opportunity to select the target answer from multiple-choice options despite being faced with the possibility of losing or gaining points depending on the correctness of their selection, participants were more likely to choose to see the multiple-choice options and were also more likely to select the correct answer if they were in a TOT state compared to a non-TOT state. These findings are consistent with other research examining knowledge gaps and information-seeking behaviors (e.g., FitzGibbon et al. 2020; Gruber et al. 2014; Kang et al. 2009; Wade and Kidd 2019), suggesting that participants should be more likely to spend limited resources when experiencing a perceived gap in knowledge, as they are curious to discover the missing piece of information.

Taken together, the aforementioned studies (Cleary et al. 2021b; Litman et al. 2005; Metcalfe et al. 2017, 2020, 2022; Rousseau and Kashur 2021; Schwartz 2002b) suggest that one possible means by which people experience a feeling of closeness to a desired piece of information that could resolve a perceived small knowledge gap is the TOT state. Moreover, these studies suggest a direct link between TOT states, curiosity, and information-seeking behaviors. This points toward the possibility that metacognitive sensations of memory that occur during retrieval failure, such as the TOT state, are a cognitive means by which curiosity and its corresponding information-seeking behavior can emerge.

### 1.4. Déjà vu-Like States

Another way in which a sensation of closeness to a piece of information that has the potential to resolve a current knowledge gap might emerge through metacognitive monitoring is through déjà vu-like states. Déjà vu is the feeling of having experienced a current situation before, while simultaneously believing that this is the first time the situation has ever been encountered (e.g., Brown 2003, 2004; Cleary and Brown 2022). Whereas, the TOT state is described as a feeling of being on the verge of retrieving an as yet unretrievable word. Cleary et al. (2019) describe the déjà vu state as possibly involving a feeling of being on the verge of recollecting from memory how a scene or scenario is going to unfold before a person (or being on the “tip of an experience,” p. 1439).

Moreover, Schwartz and Cleary (2016) proposed that, as has been proposed for TOT states, an adaptive function of both TOT and déjà vu states might be to engage in further information-seeking behaviors. That is, the déjà vu state, while seeming jarring and puzzling in the moments that it is being experienced, might serve the useful function of prompting the experiencer to direct attention and cognitive effort toward a search of memory to discover the reason for the sensation. From this perspective, the déjà vu state (and déjà vu-like states) might be expected to be associated with curiosity and information-seeking behaviors similarly to how TOTs have been shown to have such an association. Establishing such a connection would add another missing puzzle piece that will help to link the curiosity literature with the metacognition literature in the growing effort to understand the mechanisms behind curiosity. Additionally, as noted by Litman (2009), a remaining gap in the literature is attempting to link curiosity and metacognition concerns when a person fails to retrieve visual or auditory information, as research to date has only focused on instances in which the individual fails to remember the answers to general knowledge questions.

### 1.5. The Present Study

The present study examined whether déjà vu and déjà vu-like states (i.e., the auditory version of déjà vu known as déjà entendu) are associated with curiosity and information-seeking behaviors. Toward this end, the present study combined a paradigm that has been used to study reports of déjà vu (Experiment 1)—and also a paradigm that has been used to study reports of déjà entendu (Experiment 2)—with the method of examining curiosity and information-seeking behavior used by Metcalfe et al. (2017) in their study of TOT states.

#### 1.5.1. A Paradigm for Studying Déjà vu Reports

**Figure 1:**
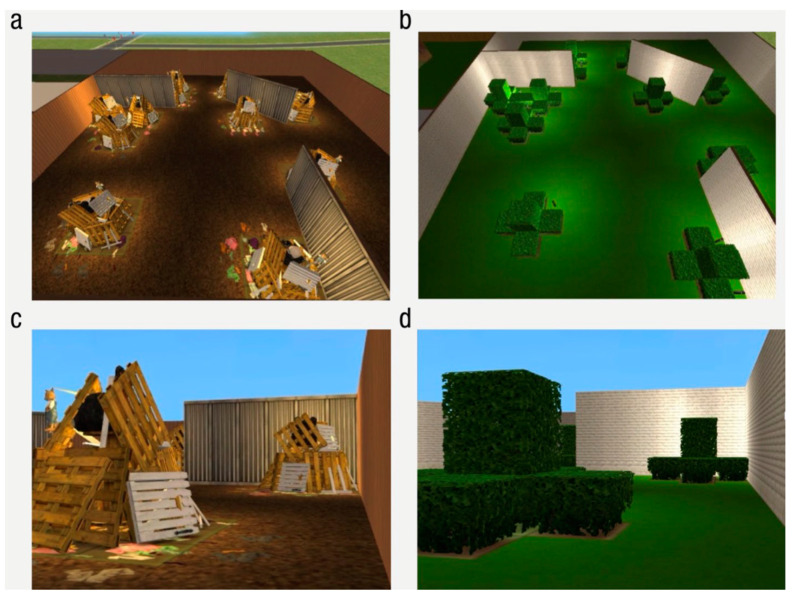
Example Scene Stimuli. From Cleary and Claxton (2018). Panels (**a**,**b**) represent a bird’s-eye view of the junkyard study scene, which spatially corresponds to the hedge garden test scene. Panels (**c**,**d**) represent the participant’s first-person perspective of these study–test pairs.

The paradigm used in Experiment 1 of the present study follows from past empirical work on déjà vu reports. In past work, researchers have used a test paradigm in which otherwise novel test scenes share spatial features with earlier viewed scenes. For example, Cleary et al. (2012) used immersive three-dimensional scenes in a virtual reality environment to examine how novel scenes that share a spatial configuration (e.g., a hedge garden scene with a particular spatial layout) with a previously viewed scene (e.g., a junkyard scene with that same spatial layout) might prompt a sense of déjà vu in cases of recall failure (see Figure 1). Participants were more likely to report déjà vu if a test scene spatially mapped onto a studied scene that failed to be recalled than if it did not. Later, Cleary and Claxton (2018) and Cleary et al. (2021a) demonstrated a similar pattern with dynamic video-based first-person virtual tours through the same scenes. This general paradigm has also shown strong associations between déjà vu reports and other metacognitive judgments, such as feelings of prediction (Cleary and Claxton 2018; Cleary et al. 2021a) and feelings of postdiction (Cleary et al. 2019).

In Experiment 1 of the present study, we searched for an association between déjà vu reports and feelings of curiosity regarding the potential experimental source of any perceived familiarity with a test scene. We also searched for an association between déjà vu reports and information-seeking behaviors. One way that we did this was to examine participant inclinations to expend limited experimental trials toward discovering the source of any perceived familiarity with a scene, analogously to what was done in Metcalfe et al.’s (2017) investigation of TOT state. Another way that we did this was to examine possible proxies for memory retrieval search effort, such as the tendency to make commission errors while trying to retrieve relevant information, which has been shown to be associated with TOT states (Huebert et al. 2023) and with increases in perceived stimulus familiarity (Carlaw et al. 2022), as well as the amount of time spent at the prompt trying to retrieve a possible source for any perceived familiarity.

#### 1.5.2. A Paradigm for Studying Déjà Entendu Reports

As previously discussed, Litman (2009) called for additional research examining the mechanisms and consequences of curiosity for auditory information, an under-investigated area. Therefore, in Experiment 2, we instead used an auditory paradigm as a means of investigating an auditory déjà vu-like experience, often termed déjà entendu. Déjà entendu is the feeling of having heard something before despite believing that it is new (Brown 2003, 2004; Cleary and Brown 2022; McNeely-White and Cleary 2019). In the first study to empirically examine déjà entendu, McNeely-White and Cleary (2019) sought to use auditory analogs to the visual scenes used in the aforementioned déjà vu experiments. Toward this end, they used Piano Puzzlers, which are re-writes of original famous modern songs, such as children’s tunes, pop-songs, or folk songs, in the style of a classical composer, such as Mozart, Chopin, or Bach, by composer Bruce Adolphe for a program on NPR in which callers attempt to identify the song and genre from the Piano Puzzler. The original song is embedded within the Piano Puzzler, such that some of the original features are intact yet masked by the chosen classical composer’s genre. For example, the familiar song “The Girl from Ipanema” might be turned into a Piano Puzzler by embedding phrases of the original melody within a novel song written in the style of Brahms. Piano Puzzlers often elicit a feeling of being on the verge of identifying the song, as illustrated by people who call into the radio program to try to identify the song, and Piano Puzzlers can be seen as auditory analogs to the juxtaposition of novel and familiar scene aspects in the aforementioned studies of déjà vu.

McNeely-White’s (2020) experiment was an auditory analog to the aforementioned scene-based déjà vu paradigm used by Cleary and colleagues (Cleary et al. 2012, 2021a; Cleary and Claxton 2018). Participants received an auditory study list with the original versions of clips of well known songs (e.g., “London Bridge”, “Pop Goes the Weasel”) before being presented with a test list of Piano Puzzlers. Half of the Piano Puzzlers corresponded to song clips that had been presented during the study, while the other half corresponded to original song clips that were not studied. Participants were asked to indicate whether they were experiencing déjà entendu for the Piano Puzzler, how familiar they found the song to be, and whether they could identify the song. Although having recently heard the original song clip during the study phase did not significantly increase the probability of reporting déjà entendu for the Piano Puzzler at test, a further analysis revealed that, when familiarity ratings provided during test song identification failure were examined as a function of both déjà entendu reports and study status (whether or not the original song clip was presented at study), a significant effect emerged. Specifically, while experiencing test song identification failure, participants were able to use feelings of familiarity to discriminate between Piano Puzzlers whose original song clips were experimentally familiarized during the study phase versus those that were not, but only during instances in which they also reported a sense of déjà entendu. This pattern of discrimination was not shown among non-déjà entendu reports.

In Experiment 2, we aimed to potentially create a stronger manipulation of auditory familiarity in order to increase the likelihood of déjà entendu reports. Specifically, because Piano Puzzlers do not allow for experimental control over the musical feature overlap between recently heard song clips and the Piano Puzzler test clips, in the present study, we aimed to have a high degree of experimental control over the degree of auditory feature overlap between the study clips and the test clips. Because the degree of feature overlap has been shown to be a major contributing factor to how familiar a test cue seems during retrieval failure (Huebert et al. 2022; McNeely-White et al. 2021; Ryals and Cleary 2012), and because this is grounded in theory regarding the purported computational mechanisms behind perceived cue familiarity (Clark and Gronlund 1996; McNeely-White et al. 2022), in Experiment 2, we used differing degrees of global auditory feature overlap between the test song clips and study song clips as our means of systematically attempting to increase perceived test song clip familiarity during retrieval failure (e.g., McNeely-White et al. 2021).

To achieve this in the present study, during the test, participants were presented with isolated tonal features from a well known song clip (e.g., “Mary Had a Little Lamb”). These were the notes from the song clip in their correct order but attached to an arbitrary rhythm (Kostic and Cleary 2009; McNeely-White et al. 2021), making the song difficult to identify, but potentially familiar-seeming. Each test song clip corresponded to an unaltered piano song clip that had been played in its original rhythm either three times throughout the study phase (Exposure 3x), once throughout the study phase (Exposure 1x), or not at all during study (Exposure 0x). Our logic was that increasing perceived test song clip familiarity during retrieval failure through increased auditory global feature matching to the study phase would increase the likelihood of reporting déjà entendu, thereby enabling us to examine the potential relationship between déjà entendu and curiosity, and between déjà entendu and the information seeking behaviors examined in Experiment 1 (i.e., (1) expenditure of limited experimental opportunities for learning the answer to whether an experimental source potentially produced any sense of familiarity and, if so, what are (2) the rate of commission errors and (3) the amount of time spent at the prompt trying to retrieve a possible experimental source of any potential familiarity).

#### 1.5.3. Resemblance of Our Methods to Berlyne’s Proposal

Note that our methods of studying déjà vu and déjà entendu are reminiscent of the aforementioned proposal put forward by Berlyne decades ago regarding curiosity (e.g., Berlyne 1950, 1960). Berlyne proposed that some forms of curiosity might emerge when the individual is presented with a novel stimulus that contains familiar elements, creating a strange juxtaposition between old and new elements. Although this specific proposal has yet to be experimentally investigated, the paradigms used to study déjà vu and déjà entendu involve this type of juxtaposition of familiarized and novel elements, and in this way, present yet another means by which the present study adds an important puzzle piece to the overall literature to help to further tighten the link between curiosity and metacognition studies.

## 2. Experiment 1

In Experiment 1, we examined whether participants reporting déjà vu for a scene would also report higher feelings of curiosity regarding the potential source of any perceived familiarity with that scene. We further examined whether participants would be more likely to use limited experimental opportunities to see the potentially corresponding study scene that shared the spatial layout of a current novel test scene when experiencing déjà vu for that scene, analogously to how Metcalfe et al. (2017) found that participants were more likely to use limited experimental opportunities to learn the answer to a general knowledge question during TOT than non-TOT reports. This was performed by allowing participants to request seeing the potential study scene that mapped onto the test scene on up to 20% of the test scene trials. In addition, we examined whether other potential indices of information-seeking behaviors could potentially be related to déjà vu, as well. Specifically, we examined whether participants would spend more time at the recall prompt during déjà vu than non-déjà vu reports (a possible indicator of retrieval search effort) and whether participants would be more likely to generate commission errors, or incorrect candidate source information, during déjà vu than non-déjà vu reports (a possible indicator of retrieval search effort), as has been shown with face familiarity (Carlaw et al. 2022) and TOT states (Huebert et al. 2023).

## 3. Method

### 3.1. Participants

Participants were 72 undergraduates from Colorado State University. Previous research examining biases that occur during déjà vu reports (Cleary and Claxton 2018) found large effect sizes (*d_z_* = 1.33 in Experiment 2 and *d_z_* = 1.54). However, as no research has yet been conducted on feelings of curiosity for these types of scene stimuli, let alone during déjà vu experiences, a conservative power analysis was used, with power set to 0.90, an α of 0.05, and a medium effect size (*d* = 0.50). G*Power (Faul et al. 2007) was used. This indicated that the necessary sample size to demonstrate such an effect would be 44 participants. Because of the nature of the experiment sign-up process, more participants than were necessary based on the power analysis completed the experiment. The experiment was conducted on the last day of the academic semester, and experiment timeslots were over-posted as a means of ensuring that enough participants would sign up, as many undergraduate students may have been busy studying for finals and, therefore, been too busy to make a given experiment timeslot work with their schedules. However, more students than expected signed up for the experiment and, instead of being turned away, were allowed to complete the experiment, and, therefore, they were included in the dataset. All participants received course credit toward an undergraduate course in return for participating in this experiment.

### 3.2. Materials

Stimuli consisted of 64 study-test pairs of configurally similar scenes that have been used in prior research examining the déjà vu phenomenon (Cleary et al. 2012; Cleary and Claxton 2018). These scenes were designed such that the test scenes contained the exact spatial layout of elements from the contextually unique study scenes (see Figure 1). In addition to using the 64 study-test pairs, audio files created by Cleary et al. (2012) were used during the study phase to indicate the name of the scene (e.g., “This is a junkyard. Junkyard” was played while participants viewed the junkyard study scene). E-Prime 3.0 was used to present the experiment. All experiment files and stimuli are available on the Open Science Framework at https://osf.io/9j82m/ (accessed on 29 July 2022).

Two counterbalanced versions of the experiment were created, such that each test scene fell into the studied and unstudied condition. For example, in the first counterbalanced version, the garden hedges test scene spatially corresponded to a scene presented at study (the junkyard study scene), while, in the second counterbalanced version, the garden hedges test scene did not spatially correspond to a scene presented during study.

### 3.3. Procedure

Participants were randomly assigned to one of the two counterbalanced versions of the experiment. The 64 study-test pairs were divided into two study-test blocks. Each study list consisted of 16 virtual tour videos each accompanied by an audio file with a female voice indicating the name of the scene. After each study list came the test list, which consisted of 32 experimentally unique virtual tour videos. Half of these randomly ordered test scenes corresponded to spatially similar studied scenes, while the other half did not.

Prior to beginning the experiment, participants were presented with instructions explaining their tasks throughout the experiment (see Supplementary File S1). After reading through the instructions, participants began the first study block. Their only task was to watch each virtual tour video carefully and try to remember the name being spoken for it. Once the study list of 16 virtual tour videos was complete, participants were then given specific instructions before completing the first test block. They were instructed that they would now view a new list of virtual tour videos, none of which would have been seen during the study phase. They were told that some of the virtual tours might remind them of a scene presented during the study phase. After each video, they would be asked a series of questions concerning the scene. First, they would be asked to indicate whether they are experiencing déjà vu, which was defined as “the feeling of having been someplace or done something before, without being able to pinpoint why, and despite knowing that the current situation is new.” Next, they would be asked to indicate how familiar the current test scene feels on a scale from zero (*Not at all familiar*) to 10 (*Extremely familiar*). Next, they would be told that they should indicate how curious they feel about the current test scene on a scale from zero (*Not at all curious*) to 10 (*Extremely curious*). They would then be prompted to indicate whether they can think of a scene from the study phase that reminds them of the current test scene, and if so, to type in the name of that scene. Finally, they would then be told that they will have limited opportunities to discover information concerning the current test scene, namely, to which study scene (if any) it corresponds. However, they would only be able to see this information on approximately 20% of the trials, which totaled six opportunities per test block.^1^

After receiving the test instructions, participants then began the first test block, consisting of 32 randomly ordered virtual tour videos, half of which corresponded to scenes presented during the study phase in their spatial layout. After watching each virtual tour video from the first-person perspective, participants were asked a series of questions concerning the test scene, with each question appearing one at a time on the screen. The first question asked participants to indicate whether they were experiencing déjà vu (Y = Yes, N = No). The second question next asked them to indicate how familiar the test scene felt on a scale of zero to 10 (*0 = Not at all familiar, 10 = Extremely familiar*). The third prompt asked participants to indicate how curious they felt about the test scene on a scale of zero to 10 (*0 = Not at all curious, 10 = Extremely curious*). A fourth question asked participants whether they could think of a scene from the study phase that reminded them of the current test scene, and if so, to type in the name of that scene. Finally, participants were then asked to indicate whether they would like to use their limited opportunities to see information about the current test scene (Y = Yes, N = No). For this question, the remaining number of opportunities was displayed at the bottom of the screen so that the participant was reminded of how many opportunities they had left (e.g., the text “3 opportunities remaining” was displayed at the bottom left-hand side of the screen), along with the trial number (e.g., the text “Trial 4/16” was displayed at the bottom right-hand side of the screen). If the participant indicated that they did indeed want to see information concerning the test scene, then a still-image of the configurally similar study scene was displayed in the middle of the screen, along with the name of the study scene. However, if the test scene did not correspond to a configurally similar study scene, then the text “This scene does NOT correspond to a scene presented at study” was displayed in the middle of the screen. To prevent participants from simply saying “No, do not use limited opportunities” as a means of completing the experiment faster, the text “Please wait…” appeared if the participant indicated “No” on this prompt, which lasted the same duration (three seconds) as if they had indicated “Yes.”

After completing the first study-test block, participants then proceeded to the second study-test block. Additionally, the limited-opportunities counter was reset, such that participants were able to use their limited resources to see information pertaining to the current test scene on up to six of the trials in the second test phase.

## 4. Results

In both Experiments 1 and 2, data were analyzed using null hypothesis significance testing (NHST) and Bayesian methods of analysis. In addition to reporting *p*-values and standard effect sizes, specifically Cohen’s *d* for repeated-measures and partial eta squared ($n_{p}^{2}$; see Lakens 2013) for parametric tests and matched-pairs rank-biserial correlation for nonparametric tests (*r_rb_*; see Kerby 2014), produced from NHST using JASP, Bayes Factors (*BF*s) are also reported; these were computed using JASP with the JZS prior, as it requires the fewest prior assumptions about the range of true effect sizes (Rouder et al. 2009). The recommendations proposed by Wagenmakers (2007) concerning strength of evidence provided by Bayes Factors were used, such that we considered a Bayes Factor to provide either anecdotal evidence (*BF* = 1–3), substantial evidence (*BF* = 3–10), strong evidence (*BF* = 10–30), very strong evidence (*BF* = 30–100), or extreme evidence (*BF* > 100) in favor of one hypothesis, either the null or alternative, over the other. A Bayes Factor of 1 was considered to provide no evidence for either the null or alternative hypothesis. In the results sections reported below, *BF_10_* was reported when arguing in favor of the alternative hypothesis while *BF_01_*, which is the reciprocal of *BF_10_*, was reported when arguing for the null hypothesis.

### 4.1. Scene Recall Rates

It is important to first consider how often participants were able to recall a studied scene that shared the same layout at a test (in cases where they shared the same spatial layout). On average, when presented with a novel test scene that had the same spatial layout as a studied scene, participants correctly identified (i.e., provided the name of the corresponding study scene or provided partially identifying information) on 26% (*SD* = 0.19) of the trials. Note that trials were hand-labeled as being either an instance of scene recall success (e.g., the participant typed in the target studied scene’s name), partial recall success (e.g., the participant typed in “wedding” for the target scene Arbor; note that only one participant throughout the experiment had a trial labeled as partial recall success), or scene recall failure (e.g., the participant typed in the incorrect target name or left the prompt blank). The trials labeled as scene recall failure were further subdivided as being an instance of a commission error (the participant typed in incorrect information) or an omission error (the participant left it blank or typed “Don’t know,” “Can’t remember,” etc.); these data are described below.

### 4.2. Replications of Previous Findings

#### 4.2.1. Probability of a Déjà vu Report during Retrieval Failure

Overall, during retrieval failure, participants reported experiencing déjà vu on an average of 27% (*SD* = 0.15) of the trials, comparable to past studies using this paradigm. In replication of prior studies (e.g., Cleary et al. 2012, 2021a; Cleary and Claxton 2018), participants were significantly more likely to report a sense of déjà vu when the test scene mapped onto an unrecalled spatially identical study scene (*M* = 0.31, *SD* = 0.17) compared to when it did not share spatial resemblance to a studied scene (*M* = 0.26, *SD* = 0.15), *t*(69) = 3.15, *SE* = 0.02, *p* = .002, *d* = 0.38, *BF_10_* = 11.60.

#### 4.2.2. Familiarity Ratings during Retrieval Failure as a Function of Spatial Layout

Additionally, in replication of prior studies, participants gave significantly higher familiarity ratings (*M* = 3.11, *SD* = 1.54) to test scenes that shared a spatial layout with an unrecalled studied scene than to test scenes that did not (*M* = 2.72, *SD* = 1.44), *t*(71) = 4.81, *SE* = 0.08, *p* < .001, *d* = 0.57, *BF_10_* = 2116.54.

#### 4.2.3. Familiarity Ratings as a Function of Déjà vu Reports

When participants reported déjà vu during retrieval failure, they provided significantly higher familiarity ratings for the test scene (*M* = 6.26, *SD* = 1.50) than when they reported non-déjà vu (*M* = 1.66, *SD* = 1.32), *t*(69) = 20.96, *SE* = 0.22, *p* < 0.001, *d* = 2.51, *BF_10_* = 2.30 × 10^28^. This replicates patterns found in prior studies.

### 4.3. Results of Primary Interest

#### 4.3.1. Curiosity Ratings as a Function of Déjà vu Reports

**Figure 2:**
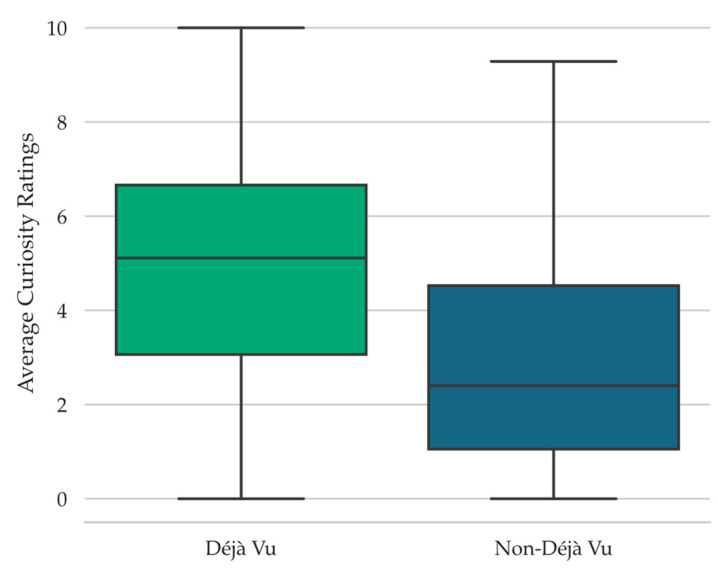
Curiosity Ratings Provided during Retrieval Failure. A boxplot of the average curiosity ratings provided during retrieval failure as a function of reported déjà vu state. Participants gave significantly higher curiosity ratings during reported déjà vu than non-déjà vu states. The boxplot follows the standard convention where the box is drawn around the first and third quartiles, the line within the box represents the median, the whiskers represent the minimum and maximum of the data distribution, and the diamonds beyond the whiskers represent outliers.

To assess the relationship between feelings of curiosity and déjà vu states, a Wilcoxon signed-rank test was conducted, examining the curiosity ratings provided during retrieval failure as a function of reported déjà vu state (note that the Wilcoxon test was conducted instead of the Student’s *t*-test because the Shapiro-Wilk test of normality was significant, *W* = 0.96, *p* = .02). When participants reported that they were experiencing déjà vu for the test scene, they provided significantly higher curiosity ratings (*Mdn* = 5.11, *Range* = 10.00) compared to when they reported a non-déjà vu state (*Mdn* = 2.40, *Range* = 9.29), *W* = 2208.00, *p* < .001, *r_rb_* = 0.88, *BF_10_* = 2.18 × 10^5^ (see Figure 2).

#### 4.3.2. Curiosity Ratings as a Function of Spatial Layout Manipulation

Although the focus of the present study is on instances of recall failure, an important consideration regarding the potential relationship between curiosity ratings and recall success vs. failure is that, based on our hypotheses, curiosity very likely drives retrieval search effort (see below). If so, then those test scene cues that elicited the greatest initial feelings of curiosity should also be more likely to be among those that eventually lead to recall success. As we do not have time course information regarding when in time curiosity feelings first emerge vs. when in time recall of the spatially mapped scene occurs, we cannot specifically examine this. However, we did find that, whereas curiosity ratings did not vary as a function of the spatial layout manipulation when only instances of retrieval failure were considered, they did when instances of recall success were included. Examining only trials on which retrieval failed, test scenes that spatially mapped onto unrecalled studied scenes did not receive higher curiosity ratings (*M* = 3.37, *SD* = 2.20) compared to those that did not (*M* = 3.26, *SD* = 2.30), *t*(71) = 1.45, *SE* = 0.08, *p* = .17, *BF_01_* = 2.86. However, when instances of recall success were included, participants had provided significantly higher curiosity ratings to test scenes containing the same spatial layout as a studied scene (*M* = 3.68, *SD* = 2.11) compared to those that did not, *t*(71) = 3.57, *SE* = 0.12, *p* < .001, *d* = 0.42, *BF_10_* = 37.19. Again, this result should be interpreted with caution given that curiosity itself may precede and even drive the retrieval search effort that ultimately leads to recall success. This possibility is particularly plausible given the below findings that curiosity was associated with indices of information-seeking and retrieval search effort, and these in turn might increase the likelihood of eventual retrieval success. Thus, it is as yet unclear if participants are most curious in situations in which they took a guess and want feedback and are curious to learn if they are correct or tend to recall more often in situations that started out with the highest levels of curiosity in the first place.

**Figure 3:**
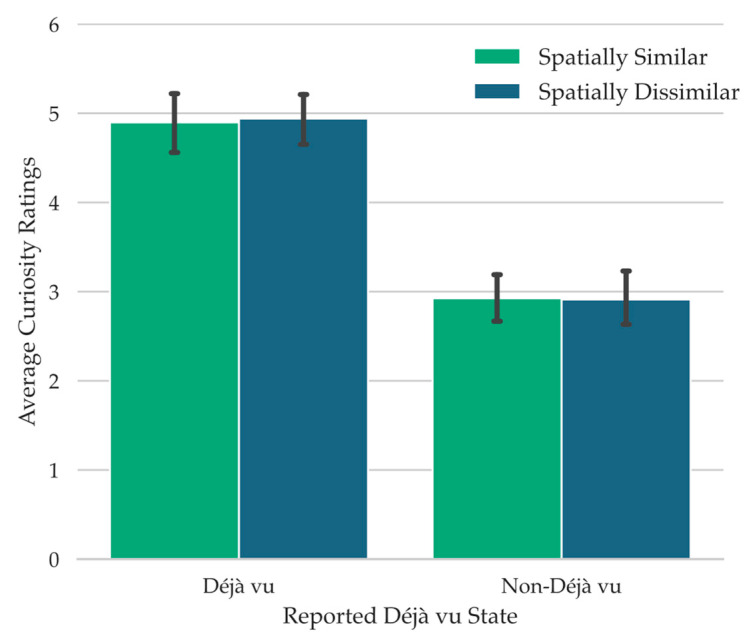
Curiosity Ratings, Spatial Similarity, and Déjà vu Reports. Average feeling of curiosity ratings as a function of spatial similarity and reported déjà vu states. Curiosity ratings were significantly higher among déjà vu reports compared to non-déjà vu reports; however, there was no difference as a function of study status. Error bars represent the standard error of the mean.

Given the association between déjà vu reports and curiosity ratings (see Figure 2), it is possible that curiosity ratings during retrieval failure follow déjà vu reports more so than the spatial layout manipulation, or that they only follow the spatial layout manipulation when déjà vu is reported. To investigate this, we carried out a 2 Spatial Similarity (Spatially Similar, Spatially Dissimilar) × 2 Déjà vu Status (Déjà vu, non-Déjà vu) repeated-measures ANOVA on the curiosity ratings provided during retrieval failure. This analysis revealed a significant main effect of Déjà vu Status, *F*(1, 67) = 57.05, *MSE* = 4.77, *p* < .001, $n_{p}^{2}$ = 0.46, *BF_10_* = 1.17 × 10^21^ (see Figure 3). There was no main effect of Spatial Similarity, *F*(1, 67) = 0.03, *MSE* = 0.62, *p* = .88, *BF_01_* = 7.38, nor a significant interaction, *F*(1, 67) = 0.08, *MSE* = 0.68, *p* = .78, *BF_01_* = 5.41. This pattern suggests that curiosity ratings largely followed déjà vu reports.

#### 4.3.3. The Relationships between Familiarity Ratings and Feelings of Curiosity

We now turn to the results pertaining to the relationship between participants’ feelings of familiarity and feelings of curiosity during retrieval failure. The correlation between familiarity and curiosity ratings during retrieval failure was computed for each participant. These correlations were then analyzed using a one-sample Wilcoxon signed-rank test with a test value of zero (note that the normality assumption was violated, with the Shapiro-Wilk test being significant, *W* = 0.96, *p* = .02). Overall, participants’ familiarity ratings (*Mdn* = 3.27, *Range* = 9.31) and curiosity ratings (*Mdn* = 2.98, *Range* = 6.27) during retrieval failure were significantly correlated with a median correlation of 0.45 (*Range* = 1.54), which was significantly greater than zero, *V* = 2317.00, *p* < .001, *r_rb_* = 0.92, *BF_10_* = 4.50 × 10^4^.

### 4.4. Information-Seeking Behaviors

#### 4.4.1. Expenditure of Limited Opportunities to Discover Source Scene

Overall, 44% of the participants used some, but not all, of their limited opportunities (*N* = 32), 51% used all of their limited resources (*N* = 37), and only 4% used none of their limited resources (*N* = 3; note that these three participants are necessarily excluded from the analyses presented below, as they did not use any of their limited resources). In further examination of the participants’ expenditure of limited opportunities, we found that 72% (*N* = 52) of the participants used at least 75% of their limited opportunities, and 85% (*N* = 61) used at least 50% of their limited opportunities. Upon analyzing resource allocation as a function of study-test block, participants used an average of 5.13 (*SD* = 2.10) resources on the first test block and an average of 4.65 (*SD* = 2.30) resources on the second test block. However, due to the experiment allowing participants to indicate “Yes, use limited resources” even when they were out of opportunities (note that during these instances, participants were shown the text “Out of limited opportunities”), 21 participants (29%) indicated “Yes, use limited resources” more than six times per test block. For example, one participant indicated “Yes, use limited resources” on eight of the test trials despite only having a total of six opportunities to actually view the corresponding study scene. These instances of wanting to expend resources that did not exist were included in the analyses reported below, as one may argue that those instances in which the participant indicated “Yes, use limited resources” despite not having any might be the most intense in terms of curiosity, familiarity, and/or déjà vu.

#### 4.4.2. Expenditure of Limited Opportunities as a Function of Déjà vu Reports

**Figure 4:**
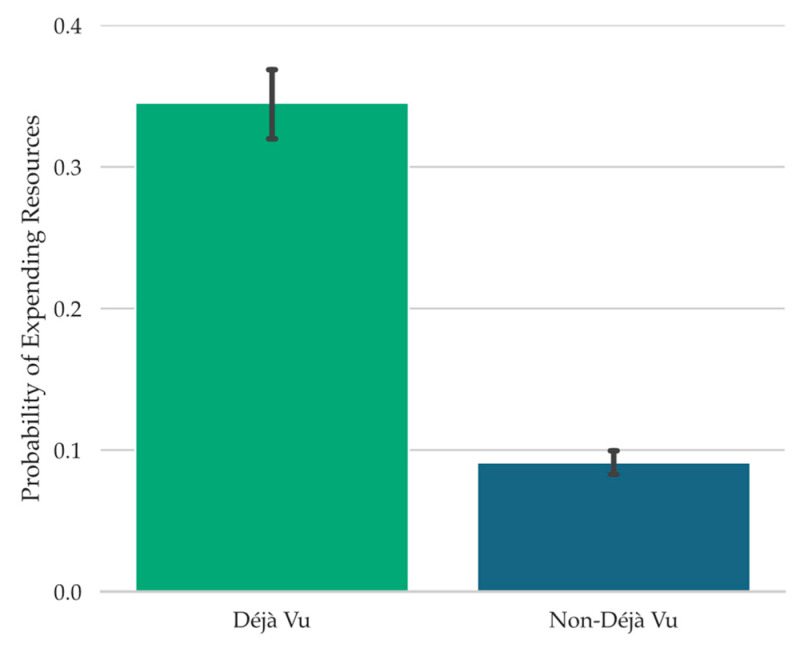
Resource Expenditure During Déjà Vu States. The average probability of participants indicating “Yes, use limited resources” as a function of reported déjà vu state. Participants were significantly more likely to expend resources while experiencing a sense of déjà vu. Error bars represent the standard error of the mean.

To examine the relationship between déjà vu reports and the probability of participants using their limited resources to receive information concerning the source of any familiarity with the current test scene, the proportions of trials on which participants decided to use their resources while concurrently reporting déjà vu versus non-déjà vu were computed. Participants were significantly more likely to indicate “Yes, use limited resources” when reporting déjà vu (*M* = 0.35, *SD* = 0.21) than when not (*M* = 0.09, *SD* = 0.07), *t*(67) = 9.07, *SE* = 0.03, *p* < .001, *d* = 1.10, *BF_10_* = 2.72 × 10^10^ (see Figure 4).

#### 4.4.3. Expenditure of Limited Opportunities as a Function of Spatial Layout

To examine how the spatial layout manipulation might have affected participants’ decisions to limited experimental resources for discovering the source of any familiarity with the test scene, we compared the probability of responding “Yes, use limited resources” among scenes that shared a spatial layout with an unrecalled scene with the probability of responding such among scenes that did not share a spatial layout with a studied scene. The likelihood of expending limited experimental resources was not significantly more likely among test scenes that mapped onto an unrecalled spatially similar study scene (*M* = 0.16, *SD* = 0.10) than among test scenes that did not (*M* = 0.14, *SD* = 0.06), *t*(68) = 1.59, *SE* = 0.01, *p* = .12, *BF_01_* = 2.29; however, note that the Bayes Factor only provides anecdotal evidence in support of the null hypothesis.

We further examined whether it made a difference if recall succeeded or failed. Because recall cannot succeed in the unstudied condition (in which the scenes do not share a layout with a studied scene), we compared the probability of using limited resources among scenes for recall succeeded and scenes for which recall failed, specifically by comparing the proportion of trials in the spatially similar condition on which identification succeeded vs. failed and was accompanied by a “Yes use limited resources” response. This proportion was significantly lower during scene recall failure (*Mdn* = 0.16, *Range* = 0.47) than during scene recall success (*Mdn* = 0.22, *Range* = 1.00), *W* = 571.50, *p* = .003, *r_rb_* = −0.43, *BF_10_* = 23.87 (note that a Wilcoxon signed-rank test was conducted due to the Shapiro-Wilk test being significant, *W* = 0.93, *p* = .002). This pattern suggests that participants were motivated to receive feedback on their identification attempts.

#### 4.4.4. Relationship between Expenditure of Limited Opportunities and Curiosity Ratings

We computed the average curiosity ratings provided during retrieval failure for trials on which participants decided to use their limited resources vs. not use their resources and compared these using a Wilcoxon signed-rank test, as the Shapiro-Wilk test was significant, *W* = 0.93, *p* < .001. Participants gave significantly higher curiosity ratings among trials on which they decided to use their limited opportunities to receive information concerning the current test scene (*Mdn* = 5.00, *Range* = 10.00) compared to when they decided against using their limited opportunities (*Mdn* = 2.82, *Range* = 9.29), *W* = 2208.00, *p* < .001, *r_rb_* = 0.94, *BF_10_* = 4.17 × 10^6^. These data are supportive of prior research suggesting that feelings of curiosity affect participants’ information-seeking behaviors (e.g., Kang et al. 2009).

#### 4.4.5. Time Spent at Retrieval Prompt

Besides the limited experimental resource expenditure described above, another way in which information seeking behaviors might manifest is in the amount of time spent trying to retrieve potentially relevant information. One indicator of this might be the amount of time spent at the Recall prompt. During instances of recall failure, specifically during instances of omission error, as commission errors inherently require more time spent on the Recall prompt to physically type out the identification attempt, participants spent significantly more time on the Recall prompt for trials on which déjà vu was reported (*Mdn* = 1239.14 ms, *Range* = 16239.00 ms) compared to when non-déjà vu was reported (*Mdn* = 1008.77 ms, *Range* = 7543.24 ms), *W* = 1599.00, *p* = .002, *r_rb_* = 0.45, *BF_10_* = 67.32 (note that the Shapiro-Wilk test of normality was significant, *W* = 0.48, *p* < .001). 

Among instances of omission error, no difference was found in the amount of time spent on the Recall prompt when the test scene did (*Mdn* = 1138.63 ms, *Range* = 7711.69 ms) versus did not (*Mdn* = 1023.42 ms, *Range* = 6316.20 ms), and they share a spatial layout with a studied scene, *W* = 1577.00, *p* = .14, *BF_01_* = 1.77 (note that the Shapiro-Wilk test of normality was significant, *W* = 0.90, *p* < .001).

#### 4.4.6. Commission Errors

**Figure 5:**
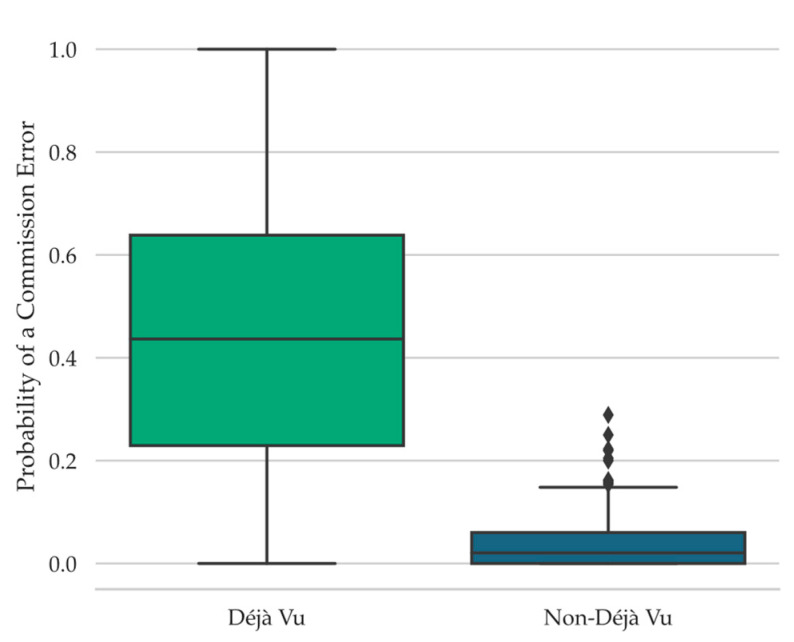
Commission Error Rates During Déjà vu Reports. A boxplot of the average probabilities of a commission error as a function of déjà vu reports. The occurrence of a commission error was significantly more likely during reported déjà vu states than non-déjà vu states. The boxplot follows the standard convention where the box is drawn around the first and third quartiles, the line within the box represents the median, the whiskers represent the minimum and maximum of the data distribution, and the diamonds beyond the whiskers represent outliers.

Emerging research suggests that metacognitive sensations of memory during retrieval failure, such as TOT states (Huebert et al. 2023) and familiarity-detection (Carlaw et al. 2022), are associated with increased efforts at conjuring up potentially relevant information in the form of making more commission errors during these metacognitive sensations of memory compared to when such sensations are absent. Therefore, as another means of assessing information-seeking behaviors during retrieval failure as a function of déjà vu reports, we examined commission errors during déjà vu compared to non-déjà vu reports. Participants were more likely to make a commission error during déjà vu (*Mdn* = 0.44, *Range* = 1.00) than non-déjà vu reports (*Mdn* = 0.02, *Range* = 0.29), *W* = 2197.00, *p* < .001, *r_rb_* = 0.99, *BF_10_* = 1.30 × 10^5^ (see Figure 5; note that the Shapiro-Wilk test of normality was significant, *W* = 0.96, *p* = .02).

Although the directionality of this relationship is not yet known, the pattern of results suggests that participants experiencing déjà vu may feel an internal drive to search for information concerning the current situation, as seen by the increased association with making a commission error. Additional research examining the time course of these processes is needed, though, to make stronger claims concerning this association.

#### 4.4.7. Commission Errors and Spatial Similarity

During retrieval failure, participants were marginally more likely to produce a commission error on trials corresponding to spatially similar study scenes (*M* = 0.18, *SD* = 0.15) compared to trials that did not correspond to spatially similar study scenes (*M* = 0.16, *SD* = 0.13), *t*(71) = 1.88, *SE* = 0.01, *p* = .06, *d* = 0.22, *BF_10_* = 0.69. Note, though, that the Bayes Factor does not provide clear evidence in favor of either the null or alternative hypothesis.

#### 4.4.8. Commission Errors and Feelings of Curiosity

To assess whether commission errors, which can be viewed as failed attempts to retrieve relevant information, are associated with increased curiosity, a Wilcoxon signed-rank test was conducted, examining whether participants provided higher curiosity ratings for trials on which they made commission errors as opposed to omission errors. Participants provided significantly higher curiosity ratings for trials on which they made commission errors (*Mdn* = 5.00, *Range* = 9.26) compared to trials on which they made omission errors (*Mdn* = 3.25, *Range* = 10.00), *W* = 241.00, *p* < .001, *r_rb_* = 0.78, *BF_10_* = 1.20 × 10^5^ (note that the Shapiro-Wilk test of normality was significant, *W* = 0.93, *p* < .001).

### 4.5. Summary of Experiment 1

Overall, the patterns obtained in Experiment 1 suggest an association between déjà vu reports and curiosity, as well as between déjà vu reports and information-seeking behaviors. Déjà vu reports were associated with higher curiosity ratings compared to non-déjà vu reports. In addition, during déjà vu reports, participants were also more likely to (1) expend limited experimental resources toward learning the potential experimental source of any test scene familiarity,^2^ (2) spend more time at the recall prompt, and (3) make more commission errors. Collectively, these results provide support for the suggestion that, similar to TOT states, déjà vu may serve the adaptive function of potentially signaling to the experiencer that they should continue to search memory for potentially relevant information (see Schwartz and Cleary 2016).

## 5. Experiment 2

The purpose of Experiment 2 was to examine how feelings of curiosity and information-seeking behaviors occur when cue information and the target information are auditory in nature. As previously discussed, there is currently a gap in the literature regarding how people experience feelings of curiosity for information that is auditory, such as music (Litman 2009). Experiment 2 examined how participants’ feelings of curiosity and information-seeking behaviors manifest when they are experiencing déjà entendu (the feeling of having heard something before despite knowing otherwise).

In Experiment 2, we also aimed to incrementally increase the level of perceived familiarity with the test items based on the global featural overlap between a given test cue and the stored memory traces corresponding to that cue. Prior research has shown that increasing the amount of global featural overlap between study and test subsequently increases participants’ perceived familiarity with the cue among both visual (e.g., Huebert et al. 2022; Ryals and Cleary 2012) and auditory (McNeely-White et al. 2021) stimuli. For example, McNeely-White et al. conducted an experiment in which participants heard isolated song rhythms (e.g., the tapped-out rhythm to “Mary Had a Little Lamb”) during the encoding phase. Each unique rhythm was played either once or three times throughout the encoding phase, thus theoretically creating either one or three memory traces containing those particular isolated rhythm features. During the test, participants were presented with the whole, unaltered versions of these songs (e.g., the piano clip containing the same rhythm that had been studied in isolation, but along with the song clip’s tonal information). Participants were significantly more likely to find the test song clip familiar if it contained an experimentally familiarized rhythm sequence compared to if it did not, and they were even more likely to find the test song familiar if its rhythm had been familiarized three times during the encoding phase than if it had only been familiarized once. The primary interest of the current study was whether this method of increasing feature familiarization might similarly increase feelings of curiosity during retrieval failure as with feelings of familiarity, and if so, if this might also affect information-seeking behaviors.

In each block of Experiment 2, participants were first presented with unaltered, whole piano song clips (e.g., “Mary Had a Little Lamb”) either once or three times at study. During the test phase, participants were then presented with isolated tonal sequences—notes occurring in the same order as their unaltered song clip but attached to an arbitrary rhythm (see Kostic and Cleary 2009; or McNeely-White et al. 2021). A third of these isolated tonal structure clips corresponded to whole song clips that were not presented at study, a third corresponded to whole song clips that were presented once at study, and a third corresponded to whole song clips that were presented three times throughout the study phase. Our primary interests were threefold: (1) would the tonal feature familiarization approach affect the likelihood of reporting déjà entendu analogously to how the spatial layout manipulation from Experiment 1 affected the likelihood of reporting déjà vu? (2) Would déjà entendu reports be associated with increased curiosity ratings? Finally, (3) would déjà entendu reports be associated with increased information-seeking behaviors? Analogously to what was done in Experiment 1 and following from Metcalfe et al. (2017), in Experiment 2, we allowed participants to indicate on up to 25% of the trials that they would like to receive information about the current test cue (this percentage was based on the overall number of trials in Experiment 2 as opposed to in Experiment 1; see note 3 below). We also examined time spent at the recall prompt and the likelihood of commission errors as other potential indices of information-seeking behaviors.

## 6. Method

### 6.1. Participants

Participants were 145 undergraduate students from Colorado State University. Three participants were lost from data analysis, though, due to either computer errors (i.e., two participants pressed the Windows key, which caused the E-Prime program to crash) or not understanding the instructions, leaving a sample size of 142. A power analysis had previously been conducted, which was based on the sample sizes used in the experiments of McNeely-White et al. (2021) and McNeely-White (2020). These prior experiments typically found small familiarity ratings and déjà entendu effect sizes when the test clip contained familiarized isolated rhythm sequences. However, it is unclear if tonal sequence cue familiarization would result in the same effect sizes as familiarizing the cue’s rhythm. A conservative power analysis was conducted using G*Power (Faul et al. 2007), with power set to 0.90, an α of 0.05, and a small effect size (*d* = 0.30), which indicated that a sample size of 119 would be sufficient to detect such an effect. As no prior research has been conducted examining feelings of curiosity and déjà entendu, we aimed to have a sample size of at least 140 participants, which was achieved.

### 6.2. Materials

The stimuli consisted of 84 of the piano song clips and their isolated tonal sequences created by Kostic and Cleary (2009) and later used by McNeely-White et al. (2021), which are all well-known pieces, such as children’s melodies and pop songs. To isolate tonal information, the notes were extracted from each song and played in their original order but according to a different, arbitrary, unstudied rhythm. All tonal sequences adhered to the same arbitrary rhythm except for a few, whose original rhythms were too similar to the arbitrary rhythm. In these instances, the tonal sequences adhered to a new, unstudied arbitrary rhythm. All experiment files and stimuli can be found on the OSF at https://osf.io/9j82m/ (accessed on 29 July 2022).

Three counterbalanced versions of the experiment were created, such that each whole, unaltered song clip fell into each of the exposure conditions across participants. For example, in the first counterbalanced version, the isolated tonal sequence test song clip “Copacabana” corresponded to a whole, unaltered song clip presented once at study (Exposure 1x). In the second version, “Copacabana” did not correspond to any whole, unaltered song clip presented at study (Exposure 0x). Finally, in the third version, “Copacabana” corresponded to a whole, unaltered song clip presented three times at study (Exposure 3x).

### 6.3. Procedure

Participants were randomly assigned to one of the three counterbalanced versions of the experiment. The 84 song segments were divided into seven study-test blocks, with each study list consisting of eight unique whole, unaltered song clip presentations. Four of these whole, unaltered song clips were presented one time throughout the study list (the Exposure 1x condition), while the other four were presented three times throughout the study list, resulting in 12 total presentations (the Exposure 3x condition). These repetitions were randomly dispersed throughout the study list, such that participants heard 16 whole, unaltered song clips at study. At test, there were 12 unique isolated tonal sequences presented, such that 1/3 corresponded to the Exposure 1x condition, 1/3 corresponded to the Exposure 3x condition, and the remaining 1/3 did not correspond to any whole, unaltered song presented during the study phase (the Exposure 0x condition). All of the isolated tonal sequences were randomly presented throughout the test list.

Prior to beginning the experiment, participants were presented with instructions explaining the experimental task (see Supplementary File S2). They also heard an example of a whole, unaltered song clip, which was not presented during the study block, and then its isolated tonal sequence. After reading through the instructions, the participants then began the first study block, which consisted of 16 whole, unaltered song clips. After listening to each song clip, they were asked to try and identify it, such as by typing in the name of the song, the lyrics, or any other information they could conjure up.

Once the study list of 16 whole, unaltered song clips was completed, participants were then given specific instructions for the first test block. They were instructed that they would now hear a new list of audio clips, but this time the audio clips would be of isolated tonal sequences, some of which would be from songs presented at study while some would not. After each isolated tonal sequence, they would be asked a series of questions. First, they would be asked to indicate whether they are experiencing déjà entendu, which was defined as “The feeling of having heard something before despite knowing it is new.” Next, they were told that they would be asked to indicate how familiar the current isolated tonal sequence feels on a scale of zero (*Not at all familiar*) to 10 (*Extremely familiar*). Next, they would be asked to indicate how curious they feel about the isolated tonal sequence on a scale of zero (*Not at all curious*) to 10 (*Extremely curious*). They would then be asked to indicate whether they can identify the isolated tonal sequence, and if so, to type in the name of the song. Finally, they were told that they would have limited opportunities to discover information concerning the isolated tonal sequence, specifically to which studied whole, unaltered song (if any) that it corresponds. However, they would only be able to use these limited opportunities on 25% of the trials.^3^ If they indicated that they did want to receive information concerning the isolated tonal sequence, then the whole, unaltered song would play along with its name being displayed on the screen. However, if there was no whole, unaltered song that was presented at study, then the text “This isolated tonal sequence does NOT correspond to a song clip heard at study” was displayed.

After receiving the test instructions, participants then began the first test block, consisting of 12 isolated tonal sequences, all of which were randomly ordered. After hearing each isolated tonal sequence, participants were asked a series of questions concerning the song fragment, with each question appearing one at a time on the screen. First, participants were asked to indicate whether they were experiencing déjà entendu (Y = Yes, N = No). The second question prompted them to indicate how familiar the isolated tonal sequence felt on a scale of zero to 10 (*0 = Not at all familiar, 10 = Extremely familiar*). The third question prompted participants to indicate how curious they felt about the isolated tonal sequence on a scale of zero to 10 (*0 = Not at all curious, 10 = Extremely curious*). The fourth question asked participants whether they could identify the name of the isolated tonal sequence, and if so, to type in that information. Finally, participants were asked to indicate whether they would like to use their limited opportunities to receive information about the current isolated tonal sequence (Y = Yes, N = No). For this question, the remaining number of opportunities was displayed at the bottom of the screen so that the participant was reminded of how many opportunities remained (e.g., “2 opportunities remaining” was displayed at the bottom left-hand side of the screen). Additionally, the trial number was also displayed so that the participant knew how many trials remained, enabling them to better manage their resources (e.g., “Trial 3/12” was displayed at the bottom right-hand side of the screen). If the participant indicated that they did indeed want to receive information concerning the isolated tonal sequence, then the whole, unaltered song began to play along with the visual text of the name of the song appearing in the middle of the screen (e.g., “A Spoonful of Sugar” appeared on the screen). However, if the isolated tonal sequence did not correspond to a whole, unaltered song presented at study, then the text “This song fragment does NOT correspond to a song clip heard at study” appeared in the middle of the screen. Finally, if the participant indicated that they did want to use their limited resources but had no more remaining, the text “Out of limited opportunities” appeared in the middle of the screen.

After completing the first study-test block, participants then proceeded to the second study-test block. Additionally, the limited-opportunities counter was reset, such that participants were able to use their limited resources to receive information pertaining to the current test song clip on 25% of the current test list’s trials. The same procedure was used for all seven study-test blocks.

Note that due to a programming error, participants in the second version of the experiment received the incorrect study list for the fifth study-test block. Therefore, this study-test block for these 31 participants was excluded from data analysis. Once the mistake was caught, the program was corrected and all subsequent participants in version two received the correct study list for the fifth study-test block.

## 7. Results

### 7.1. Song Identification Rates

Trials were hand-labeled as being either an instance of identification success (e.g., the participant typed in the correct target), partial identification success (e.g., the participant typed in “classical lullaby” for the song “Rockabye Baby”), or identification failure (e.g., they provided the incorrect song’s name or left the prompt blank). The trials labeled as identification failure were further subdivided as being an instance of a commission error (the participant typed in incorrect information) or an omission error (the participant did not type in anything or typed “don’t know,” “can’t remember,” etc.).

On average, participants identified, either fully or partially, 22% (*SD* = 0.11) of the whole, unaltered songs presented at study and 9% (*SD* = 0.07) of the isolated tonal sequences presented at test. In examining the identification rates for songs presented at study, participants were significantly more likely to identify the whole, unaltered study song if it was presented three times as opposed to only once, *t*(141) = 5.88, *SE* = 0.01, *p* < .001, *d* = 0.49, *BF_10_* = 3.65 × 10^5^ (see Table 1 for descriptive statistics). This pattern of is similar to that found by McNeely-White et al. (2021), who also demonstrated that participants are more likely to identify songs presented three times throughout the study list as there are more opportunities for identification success to occur compared to when the song is only presented once.

Turning to the identification rates for isolated tonal sequences presented during the test phase, regardless of the identification status at study, a one-way ANOVA on Exposure Condition was conducted. Because Mauchly’s test indicated a violation of the sphericity assumption, χ^2^(2) = 24.76, *p* < .001, we report Huynh-Feldt corrected results (ε = 0.87), *F*(1.74, 245.39) = 120.39, *MSE* = 0.01, *p* < .001, $n_{p}^{2}$ = 0.46, *BF_10_* = 2.83 × 10^35^. When participants were presented with an isolated tonal test sequence that was previously presented one time as a whole, unaltered song clip during the study phase, they were significantly more likely to identify it than if it had not been previously presented during study as a whole, unaltered song, as indicated by a Wilcoxon signed-rank test, *W* = 230, *p* < .001, *r_rb_* = 0.93, *BF_10_* = 1.58 × 10^6^ (note that the Wilcoxon singed-rank test was conducted due to a violation of normality based on the Shapiro-Wilk test, *W* = 0.89, *p* < .001; see Table 1 for descriptive statistics). Further, if the isolated tonal test sequence had been previously presented as a whole, unaltered song three times during the study phase, participants were significantly more likely to identify it than if it had only been presented once, *W* = 881.50, *p* < .001, *r_rb_* = 0.71, *BF_10_* = 1.63 × 10^5^ (note that we report the Wilcoxon signed-rank test results due to the Shapiro-Wilk test of normality being significant, *W* = 0.94, *p* < .001).

Collectively, it should be noted that the overall identification rates of songs at study and test in the current experiment are lower than those found in prior research using the same stimulus set. Kostic and Cleary (2009) found that participants in Experiment 2 (isolated pitch) had an average successful identification rate of whole, unaltered songs at study of 0.40 (*SD* = 0.12). At test, when presented with test songs containing experimentally familiarized tonal sequences, participants successfully identified an average of 0.21 (*SD* = 0.11) while only successfully identifying an average of 0.03 (*SD* = 0.04) of the test songs that did not contain experimentally familiarized tonal sequences. These rates are notably lower than those found in the current experiment. In Supplementary file S4, we provide a discussion of potential reasons for why the present identifications rates are lower than those found by Kostic and Cleary, along with additional analyses in which we further conditionalize based on identification status at study *and* test following from the analyses reported in the main Results section below, which only focus on instances of retrieval failure at test regardless of identification status at study. Unless otherwise noted, the patterns of results discussed below, and the more stringent results discussed in Supplementary File S4, were the same.

### 7.2. Déjà Entendu Reports

When failing to identify, either fully or partially, the isolated tonal test sequence, participants reported experiencing a sense of déjà entendu on 45% (*SD* = 0.20) of the trials. Note that this is much higher than the likelihood of reporting déjà vu in Experiment 1 but is comparable to the déjà entendu rates reported in the only published study thus far on déjà entendu (McNeely-White and Cleary 2019).

#### 7.2.1. Probability of Déjà Entendu Given Feature Familiarization

**Figure 6:**
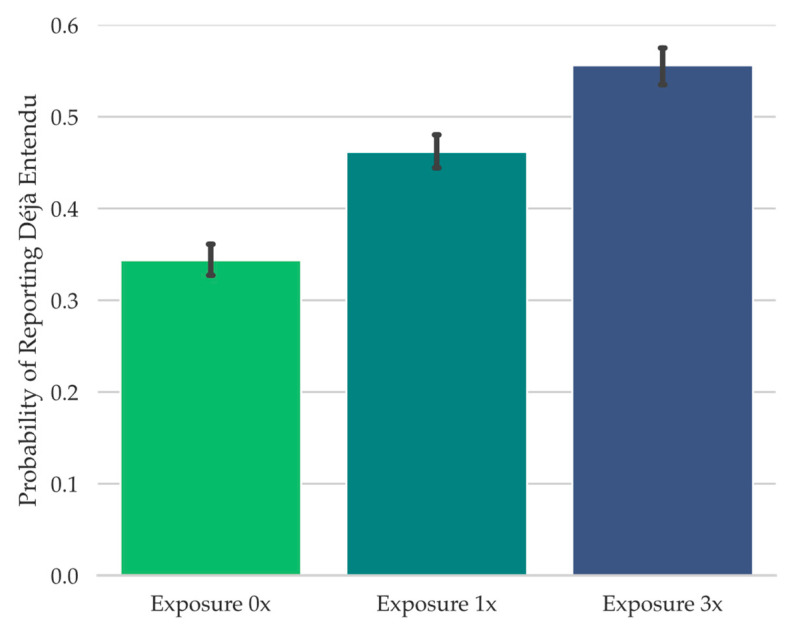
Déjà Entendu Frequency and Feature Familiarization. The average probability of reporting déjà entendu during test phase retrieval failure as a function of exposure condition. As exposure to the whole, unaltered song at study increased, so did the probability that participants reported experiencing a sense of déjà entendu for an unidentified isolated tonal test sequence. Error bars represent the standard error of the mean.

A one-way repeated-measures ANOVA examining the influence of Exposure Condition (Exposure 0x, Exposure 1x, Exposure 3x) on the probability of reporting déjà entendu for an unidentified isolated tonal test sequence was conducted. Mauchly’s test of sphericity (ε = 0.94) indicated a violation, χ^2^(2) = 11.54, *p* = .003, and we therefore report the Huynh-Feldt corrected results, which suggest a significant effect of Exposure Condition, *F*(1.88, 264.66), *MSE* = 0.01, *p* < .001, $n_{p}^{2}$ = 0.51, *BF_10_* = 3.39 × 10^40^ (see Figure 6). An unidentified isolated tonal test sequence that corresponded to a whole, unaltered song clip presented at once at study led to a greater likelihood of reporting a sense of déjà entendu (*M* = 0.46, *SD* = 0.22) compared to when the unidentified isolated tonal test sequence did not correspond to any whole, unaltered song from the study phase (*M* = 0.34, *SD* = 0.20), *t*(141) = 10.50, *SE* = 0.01, *p* < .001, *d* = 0.88, *BF_10_* = 2.44 × 10^16^. Further, when the unidentified isolated tonal test sequence corresponded to a whole unaltered song clip presented during three separate instances during the study phase, participants were significantly more likely to report a sense of déjà entendu (*M* = 0.56, *SD* = 0.25) compared to if there was only one exposure instance during the study phase, *t*(141) = 7.90, *SE* = 0.01, *p* < .001, *d* = 0.66, *BF_10_* = 1.02 × 10^10^. Note that this specific pattern of results became non-significant when further conditionalizing based on successful identification at study (see Supplementary File S4). However, the means are in the predicted direction and the Bayes Factor only provides anecdotal evidence in favor of the null hypothesis.

#### 7.2.2. Familiarity Ratings during Retrieval Failure as a Function of Feature Familiarization

**Figure 7:**
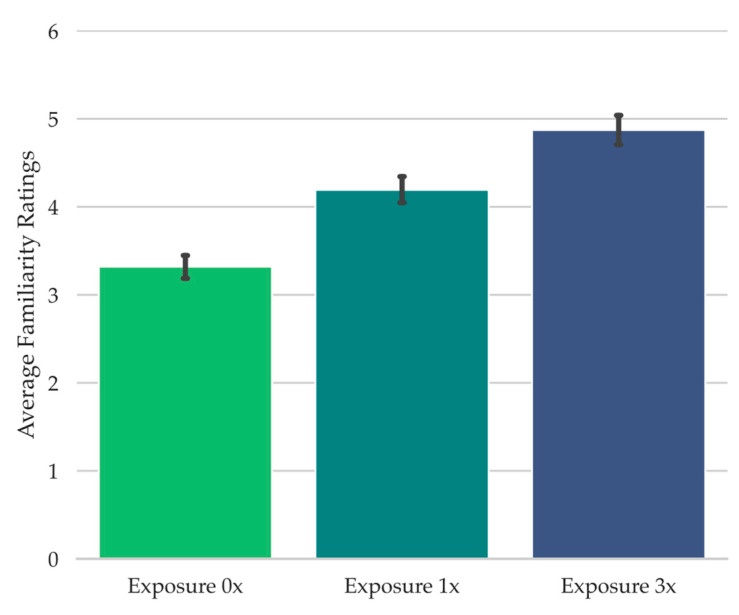
Familiarity Ratings and Feature Familiarization. Average familiarity ratings provided during test song retrieval failure as a function of exposure condition. Participants provided significantly higher familiarity ratings to unidentified isolated tonal test sequences as prior exposure to the original whole, unaltered song at study increased. Error bars represent standard error of the mean.

Familiarity ratings during test song retrieval failure mirrored the déjà entendu probabilities. The same one-way repeated-measures ANOVA performed on familiarity ratings given to unidentified isolated tonal test sequences revealed a significant effect, *F*(1.82, 256.91) = 201.81, *MSE* = 0.47, *p* < .001, $n_{p}^{2}$ = 0.59, *BF_10_* = 2.57 × 10^51^ (see Figure 7). Note that the repeated-measures ANOVA reflects a Huynh-Feldt correction, as sphericity (ε = 0.91) was violated, based on Mauchly’s test, χ^2^(2) = 16.47, *p* < .001. When presented with an unidentified isolated tonal test sequence that corresponded to a whole, unaltered song presented once during the study phase (*M* = 4.19, *SD* = 1.77), participants provided significantly higher familiarity ratings compared to when the unidentified isolated tonal test sequence did not correspond to any whole, unaltered study song (*M* = 3.32, *SD* = 1.61), *t*(141) = 13.04, *SE* = 0.07, *p* < .001, *d* = 1.09, *BF_10_* = 6.98 × 10^22^. Further, participants provided significantly higher familiarity ratings during retrieval failure for isolated tonal test sequences that corresponded to a whole, unaltered song presented three times at study (*M* = 4.87, *SD* = 2.00) compared to those that only corresponded to a whole, unaltered song clip presented once at study, *t*(141) = 9.05, *SE* = 0.08, *p* < .001, *d* = 0.76, *BF_10_* = 5.92 × 10^12^.

### 7.3. Curiosity Ratings

#### 7.3.1. Curiosity Ratings as a Function of Déjà Entendu Reports

**Figure 8:**
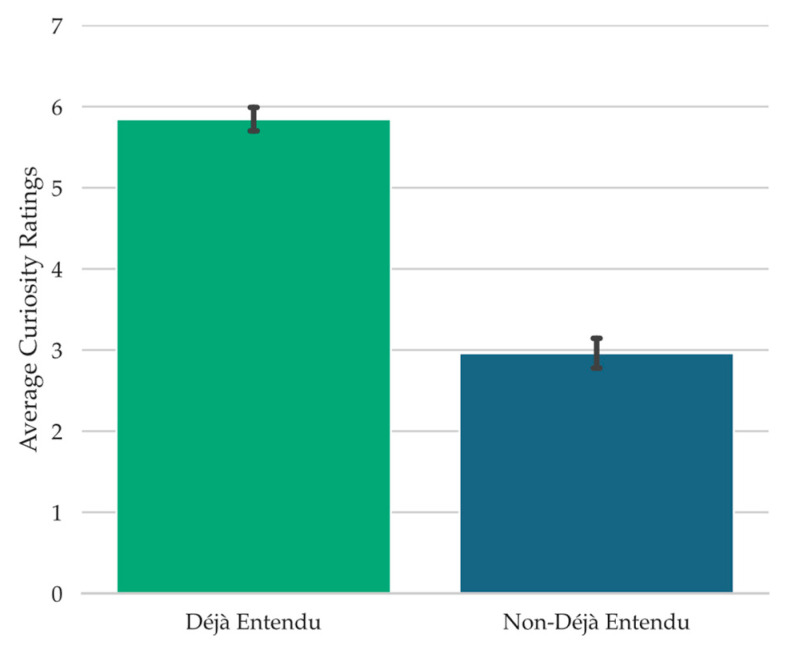
Curiosity Ratings and Déjà Entendu Reports. Average curiosity ratings provided during test song retrieval failure as a function of reported déjà entendu state. Participants provided significantly higher curiosity ratings for trials on which they also experienced a sense of déjà entendu compared to non-déjà entendu. Error bars represent the standard error of the mean.

The patterns of results in Experiment 1 suggested that when participants report feeling a sense of déjà vu, they also provide significantly higher curiosity ratings compared to when they do not report feeling a sense of déjà vu. To assess whether a similar pattern would emerge in the current experiment, a paired-samples *t*-test was conducted comparing the curiosity ratings provided to unidentified isolated tonal test sequences as a function of whether the participant reported a sense of déjà entendu versus non-déjà entendu. A similar pattern to that obtained in Experiment 1 emerged. When participants reported a sense of déjà entendu for the unidentified isolated tonal test sequence, they provided significantly higher curiosity ratings (*M* = 5.84, *SD* = 1.81) compared to when they did not report a sense of déjà entendu (*M* = 2.96, *SD* = 2.15), *t*(141) = 18.22, *SE* = 0.16, *p* < .001, *d* = 1.53, *BF_10_* = 3.46 × 10^35^ (see Figure 8).

#### 7.3.2. Curiosity Ratings as a Function of Song Feature Familiarization Manipulation

**Figure 9:**
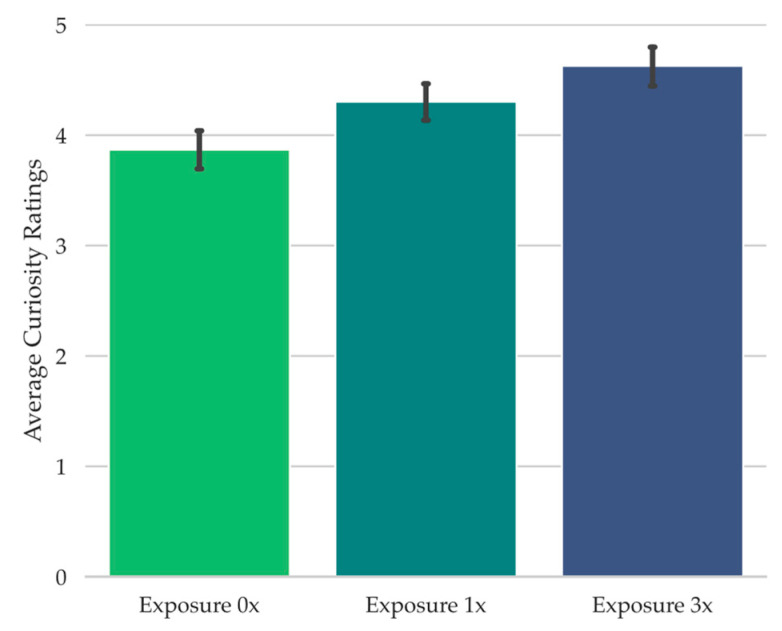
Curiosity Ratings and Feature Familiarization. Average curiosity ratings provided to unidentified isolated tonal sequences as a function of exposure condition. Participants provided significantly higher curiosity ratings to unidentified isolated tonal test sequences as the prior experimental manipulation of exposure increased. Error bars represent the standard error of the mean.

A one-way repeated-measures ANOVA performed on curiosity ratings given to tonal sequence test song clips during retrieval failure revealed a significant overall effect of feature familiarization, *F*(2, 282) = 52.98, *MSE* = 0.37, *p* < .001, $n_{p}^{2}$ = 0.27, *BF_10_* = 1.44 × 10^17^ (see Figure 9). Higher curiosity ratings were given to unidentified test song clips that corresponded to whole, unaltered song clips presented once during the study phase (*Mdn* = 4.32, *Range* = 9.50) than to unidentified test clips that did not correspond to any whole, unaltered song presented during the study phase (*Mdn* = 3.89, *Range* = 9.68), *W* = 1820.50, *p* < .001, *r_rb_* = 0.61, *BF_10_ =* 4.44 × 10^5^ (note that the Shapiro-Wilk test of normality was significant, *W* = 0.95, *p* < .001). Additionally, unidentified test song clips corresponding to an unaltered song presented three separate times at study, received significantly higher curiosity ratings (*M* = 4.58, *SD* = 2.09) than unidentified clips corresponding to a whole, unaltered song presented only once at study (*M* = 4.24, *SD* = 2.07), *t*(141) = 4.71, *SE* = 0.07, *p* < .001, *d* = 0.16, *BF_10_* = 2.30 × 10^3^. Note that when further conditionalizing based on successful identification at study, this analysis became only marginally significant (see Supplementary File S4). However, the results are in the predicted direction, and the Bayes Factor only provides anecdotal evidence in favor of the null hypothesis.

Collectively, these patterns of results provide support for Berlyne’s (1950, 1960) relative novelty proposal, as the results suggest that a novel musical piece corresponding to previously familiarized features prompts stronger feelings of curiosity than if the whole situation were novel. Further, as exposure to those previously encountered musical features increases, which thus increases the number of stored memory traces, this creates a stronger match between the current novel musical piece’s features and those stored in memory, which seems to also increase the perceived levels of curiosity. These findings also emerge when participants experience retrieval failure, in that they cannot identify the current musical piece, but they are able to sense that elements or features of the current piece correspond to information held within memory. These findings are novel, as they suggest that some forms of curiosity, specifically curiosity as a feeling-of-closeness, may emerge due to internal feature-matching processes.

In Experiment 1, the relationships between déjà vu reports, curiosity ratings, and the spatial layout manipulation were investigated, with the déjà vu reports being found to be the primary influencers of curiosity. To address whether curiosity ratings would similarly follow déjà entendu reports in the current experiment, a 2 Déjà Entendu Reports (Déjà Entendu, Non-Déjà Entendu) × 3 Feature Familiarization (Exposure 0x, Exposure 1x, Exposure 3x) repeated-measures ANOVA was conducted on participants’ curiosity ratings provided to unidentified isolated tonal test sequences. Overall, a significant main effect of Déjà Entendu Reports was found, *F*(1, 133) = 334.91, *MSE* = 4.88, *p* < .001, $n_{p}^{2}$ = 0.72, *BF*_10_ = 5.12 × 101^41^, with participants overall providing significantly higher curiosity ratings during instances of déjà entendu compared to non-déjà entendu, regardless of feature familiarization. A significant interaction was also found (note that Mauchly’s test of sphericity was significant, with an ε of 0.93, χ^2^(2) = 12.03, *p* = .002. We therefore applied the Huynh-Feldt correction), *F*(1.86, 247.87) = 10.01, *MSE* = 0.57, *p* < 0.001, $n_{p}^{2}$ = 0.07, *BF_10_* = 0.86, such that the influence of Feature Familiarization, which was also a significant main effect, *F*(2, 266) = 4.86, *MSE* = 0.61, *p* = .01, $n_{p}^{2}$ = 0.04, *BF_10_* = 0.03 (Mauchly’s test of sphericity was also violated with an ε of 0.97, χ^2^(2) = 6.75, *p* = .03, resulting in the Huynh-Feldt correction being applied; note that the Bayes Factors do not provide compelling evidence in favor of the alternative hypothesis for either the interaction or the main effect of Feature Familiarization), primarily affected curiosity ratings during déjà entendu reports. When participants reported a sense of déjà entendu for an unidentified isolated tonal test sequence, they provided significantly higher curiosity ratings if the test cue corresponded to a whole, unaltered song presented once at study (*Mdn* = 6.09, *Range* = 10.00) compared those that did not correspond to any whole, unaltered song presented at study (*Mdn* = 5.57, *Range* = 9.59), *W* = 2766.50, *p* < .001, *r_rb_* = 0.34, *BF_10_* = 31.45 (note that the normality assumption was violated, *W* = 0.87, *p* < .001). However, there was no significant increase in curiosity ratings when exposure to the whole, unaltered song at study was increased from one exposure to three separate exposures (*Mdn* = 6.40, *Range* = 9.64), *W* = 3848.50, *p* = .34, *BF_01_* = 8.41 (note that Shapiro-Wilk’s test for normality was significant, *W* = 0.93, *p* < .001). Further, when comparing the curiosity ratings provided during non-déjà entendu, no significant differences were found as a function of Feature Familiarization. Collectively, these results suggest, similarly to those found in Experiment 1, that curiosity ratings mainly followed déjà entendu reports, especially those reports that corresponded to test trials of the higher feature exposure conditions.

#### 7.3.3. The Relationship between Familiarity Ratings and Curiosity Ratings

To examine the relationship between participants’ familiarity and curiosity ratings provided to unidentified isolated tonal test sequences, the correlation between the two ratings was computed for each participant and compared against a critical value of 0 using a one-samples Wilcoxon signed-rank test. Participants provided an average familiarity rating of 4.08 (*SD* = 1.70, *Mdn* = 4.34, *Range* = 7.27) and an average curiosity rating of 4.20 (*SD* = 2.04, *Mdn* = 4.24, *Range* = 9.48). These two measures were significantly and positively correlated, with an average correlation value of 0.61 (*SD* = 0.32, *Mdn* = 0.70, *Range* = 1.50) that differed significantly from zero, *V* = 9589.00, *p* < .001, *r_rb_* = 0.97, *BF_10_* = 1.03 × 10^6^ (the Shapiro-Wilk test indicated a violation of normality, *W* = 0.86, *p* < .001). This suggests that, as the perceived level of familiarity with the unidentified test song clip increases, so does the perceive level of curiosity (although note that the direction of causality is unknown, and it could be that as curiosity increases, so does the perceived level of familiarity).

### 7.4. Information-Seeking Behaviors

#### 7.4.1. Expenditure of Limited Opportunities to Discover the Song’s Identity

We now turn to the data concerning participants’ information-seeking behaviors. Overall, 56% of the participants used some, but not all, of their limited opportunities to hear the corresponding whole, unaltered study song (*N* = 79), 40% used all of their limited opportunities (*N* = 57), and only 4% used none of their limited opportunities across all study-test blocks (*N* = 6; note that these six participants were necessarily excluded from the analyses reported below). On average, participants used 2.44 (*SD* = 1.13) of their limited resources on each of the seven study-test blocks.^4^ As was found in Experiment 1, there were some participants who indicated “Yes, use limited resources” even when they had already expended their three opportunities on the given test block. Specifically, 73 participants (51%) indicated “Yes, use limited resources” more than three times per test block (note that, like in Experiment 1, these instances were met with the displayed text “Out of limited opportunities”). For example, one participant indicated “Yes, use limited resources” on five of the test trials despite only having a total of three opportunities to actually hear the corresponding whole, unaltered study song. These instances were included in the analyses reported below, as was done in Experiment 1.

#### 7.4.2. Expenditure of Limited Opportunities as a Function of Déjà Entendu Reports 

**Figure 10:**
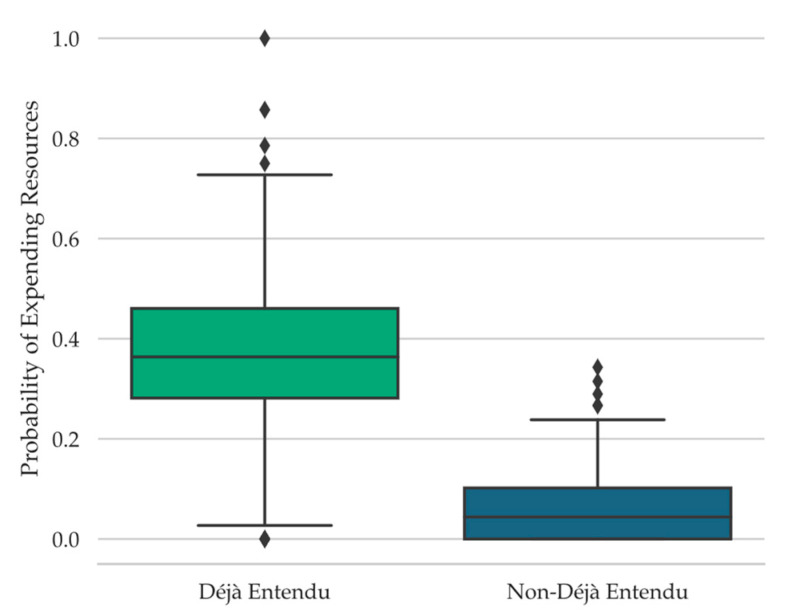
Resource Expenditure and Déjà Entendu Reports. A boxplot of the average probabilities of participants choosing to expend their limited resources as a function of reported déjà entendu state during test song retrieval failure. Participants were significantly more likely to use their resources during déjà entendu states than non-déjà entendu states. The boxplot follows the standard convention where the box is drawn around the first and third quartiles, the line within the box represents the median, the whiskers represent the minimum and maximum of the data distribution, and the diamonds beyond the whiskers represent outliers.

Based on the patterns of results found in the current experiment, specifically those demonstrating the significant association between participants’ feelings of curiosity and déjà entendu reports, there is reason to expect that participants experiencing retrieval failure during the test phase would be more likely to use their limited resources during reported déjà entendu states than non-déjà entendu states, as this would be an outward manifestation of their internal metacognitive feelings. As was shown in Experiment 1, participants were indeed more likely to use their limited resources while experiencing déjà vu than non-déjà vu. To determine whether such an effect would emerge in the current experiment assessing curiosity for musical stimuli, a Wilcoxon signed-rank test was conducted, comparing the probability that participants experiencing déjà entendu would indicate “Yes, use limited resources” against the probability of them saying “Yes, use limited resources” while experiencing non-déjà entendu. Indeed, a significant effect emerged, such that the probability of indicating “Yes, use limited resources” was significantly higher on trials associated with déjà entendu (*Mdn* = 0.36, *Range* = 1.00) compared to non-déjà entendu (*Mdn* = 0.04, *Range* = 0.34), *W* = 9116.00, *p* < .001, *r_rb_* = 0.99, *BF_10_* = 3.08 × 10^8^ (see Figure 10; note that the Shapiro-Wilk’s test for normality was significant, *W* = 0.98, *p* = .01).

#### 7.4.3. Expenditure of Limited Opportunities as a Function of Feature Familiarization

**Figure 11:**
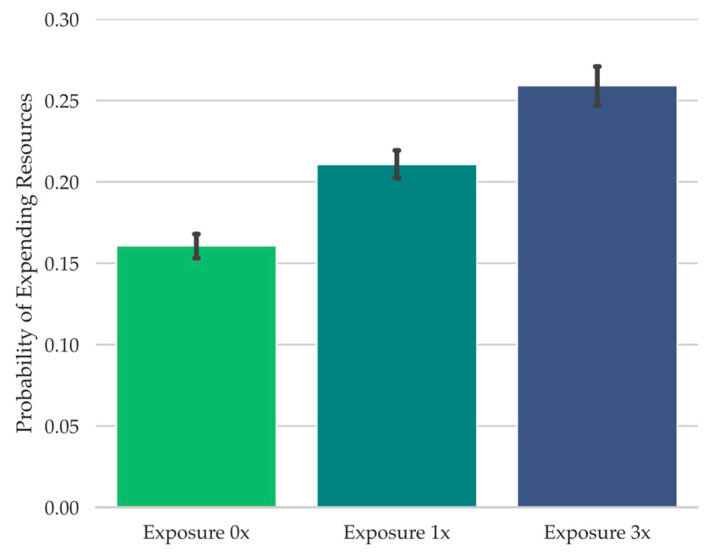
Expenditure of Limited Opportunities and Feature Familiarization. Average probability of participants using limited resources as a function of exposure condition, during test song retrieval failure. Participants were significantly more likely to use their limited resources when an unidentified isolated tonal sequence corresponded to whole, unaltered song(s) presented at study. Error bars represent standard error of the mean.

To examine whether the song clip feature familiarization affected how participants allocated their limited resources to discover the identity of the song corresponding to the current isolated tonal test sequence, the proportions of trials on which participants indicated “Yes, use limited resources” were examined among instances of test song identification failure as a function of the song clip feature familiarization manipulation. A one-way repeated-measures ANOVA revealed a significant main effect of exposure condition on the probability that participants experiencing test song retrieval failure would indicate “Yes, use limited resources”, *F*(2, 268) = 38.27, *MSE* = 0.01, *p* < .001, $n_{p}^{2}$ = 0.22, *BF_10_* = 4.17 × 10^12^. As can be seen in Figure 11 below, participants were significantly more likely to indicate “Yes, use limited resources” for unidentified isolated tonal test sequences that corresponded to a whole, unaltered song presented once during the study phase (*M* = 0.21, *SD* = 0.10) compared to those that did not correspond to any whole, unaltered study songs (*M* = 0.16, *SD* = 0.09), *t*(134) = 4.94, *SE* = 0.01, *p* < .001, *d* = 0.43, *BF_10_* = 5.62 × 10^3^. When exposure to the whole, unaltered song was increased from one instance to three separate instances during the study phase, participants were even more likely to indicate “Yes, use limited resources” to receive information concerning the unidentified isolated tonal test sequence (*M* = 0.26, *SD* = 0.13), *t*(134) = 4.19, *SE* = 0.01, *p* < .001, *d* = 0.36, *BF_10_* = 314.51. However, note that this pattern of results became non-significant when conditionalizing based on successful song identification at study (see Supplementary File S4).

Like in Experiment 1, the trials on which identification succeeded was significantly more likely to be accompanied by a “Yes” response (*Mdn* = 0.29, *Range* = 1.00) than trials on which identification failed (*Mdn* = 0.22, *Range* = 0.34), *W* = 6405.50, *p* < .001, *r_rb_* = 0.42, *BF_10_* = 1.15 × 10^3^ (note that a Wilcoxon signed-rank test is reported due to the Shapiro-Wilk test of normality being significant, *W* = 0.97, *p* = .002). As in Experiment 1, this may be suggesting that participants were motivated to receive confirmatory feedback on their identification attempts that they were uncertain about.

#### 7.4.4. Relationship between Expenditure of Limited Opportunities and Curiosity Ratings

The patterns of results in Experiment 1 suggested a significant relationship between participants’ resource allocation behaviors and subjective curiosity ratings, such that they provided significantly higher curiosity ratings for trials on which they decided to use their limited resources to discover information about the test scene while experience retrieval failure. To determine whether a similar pattern of behavior would emerge in the current experiment, a paired-samples *t*-test was conducted on participants’ subjective curiosity ratings provided during test song retrieval failure as a function of whether they decided to use or save their limited resources. Indeed, a similar pattern did emerge. Participants provided significantly higher curiosity ratings on trials they decided to expend their limited resources (*M* = 6.62, *SD* = 1.92) compared to trials on which they decided to save their limited resources (*M* = 3.74, *SD* = 2.06), *t*(134) = 16.92, *SE* = 0.17, *p* < .001, *d* = 1.46, *BF_10_* = 6.18 × 10^31^.

#### 7.4.5. Relationship between Expenditure of Limited Opportunities and Familiarity Ratings

To assess how subjective feelings of familiarity correlated with participants’ decisions to either use or save their limited resources, a paired-samples *t*-test was conducted. Overall, when participants failed to identify the isolated tonal test sequence, they provided significantly higher familiarity ratings when they also decided to use their resources (*M* = 6.47, *SD* = 1.94) compared to when they decided to save their resources (*M* = 3.55, *SD* = 1.61), *t*(134) = 20.30, *SE* = 0.14, *p* < .001, *d* = 1.75, *BF_10_* = 2.16 × 10^39^.

#### 7.4.6. Time Spent at the Retrieval Prompt

As previously discussed, one manifestation of increased information-seeking behaviors during retrieval failure might be increased memory search times, as the participant may be internally searching for potentially relevant information stored within memory. Such an effect was found in Experiment 1, with participants spending longer on the Recall prompt while experiencing déjà vu than non-déjà vu, specifically while making omission errors. A similar effect was found in Experiment 2. Examination of the reaction time data, as measured by the amount of time participants remained on the Recall prompt before proceeding, revealed a significant difference as a function of reported déjà entendu state. Specifically, based on a Wilcoxon signed-rank test, on trials associated with a déjà entendu report and an omission error, participants spent a significantly longer amount of time on the Recall prompt (*Mdn* = 1362.52 ms, *Range* = 4135.15 ms) compared to trials associated with a non-déjà entendu report (*Mdn* = 1210.40 ms, *Range* = 4102.63 ms), *W* = 7252.00, *p* < .001, *r_rb_* = 0.47, *BF_10_* = 1.57 × 10^3^ (note that the Shapiro-Wilk test was significant, *W* = 0.91, *p* < .001). These findings are similar to those of Experiment 1, such that the presence of a déjà entendu state might encourage the experiencer to spend more time searching for relevant information as to why they are experiencing this strange metacognitive sensation.

As was found in Experiment 1, among instances of omission error, no differences were found in the amount of time spent on the Recall prompt when the unidentified isolated tonal test sequence varied in the level of prior feature familiarization at study, *F*(1.74, 243.13) = 2.10, *MSE* = 155452.81, *p* = .13, *BF_01_* = 5.52 (note that Mauchly’s test of sphericity was significant, with a ε of 0.87, χ^2^(2) = 24.99, *p* < .001. The reported ANOVA reflects the Huynh-Feldt correction.).

#### 7.4.7. Commission Errors

**Figure 12:**
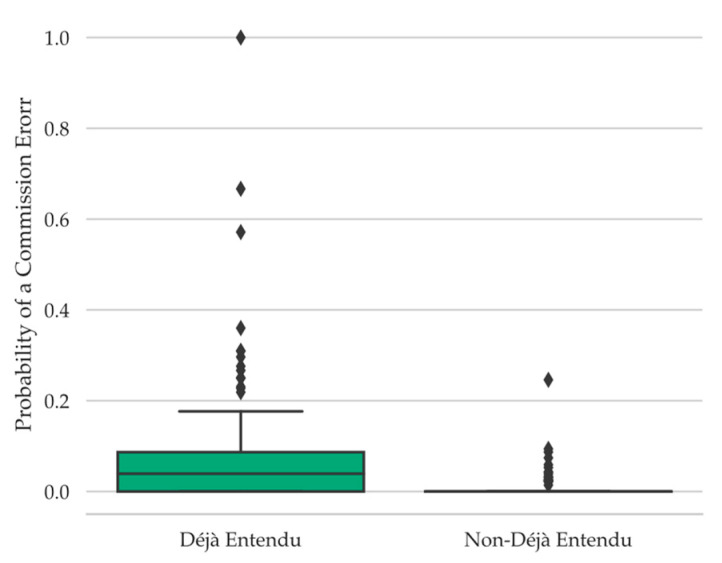
Commission Error Rates During Déjà Entendu Reports. A boxplot of the average probabilities of a commission error as a function of déjà entendu reports. The occurrence of a commission error was significantly more likely during reported déjà entendu states than non-déjà entendu states. The boxplot follows the standard convention where the box is drawn around the first and third quartiles, the line within the box represents the median, the whiskers represent the minimum and maximum of the data distribution, and the diamonds beyond the whiskers represent outliers.

Experiment 1 demonstrated an association between commission errors and déjà vu states, such that participants were more likely to report experiencing déjà vu on trials associated with commission errors than to report non-déjà vu, which suggests that the presence of a retrieval-failure-based metacognitive state might encourage increased internally memory search. A similar pattern was found in the current experiment, such that test trials labeled as a commission error were more likely to be associated with déjà entendu reports (*Mdn* = 0.04, *Range* = 1.00) compared to non-déjà entendu reports (*Mdn* = 0.00, *Range* = 0.25), *W* = 5019.50, *p* < .001, *r_rb_* = 0.99, *BF_10_* = 1.06 × 10^6^ (see Figure 12; note that the Shapiro-Wilk test of normality was significant, *W* = 0.58, *p* < .001). When limiting this analysis to include only participants who did indeed make a commission error (41 participants did not make any commission errors during the test phase, and they were therefore lost from this analysis), the pattern of results was the same. For participants who at some point made a commission error during the test phase, these commission errors were more likely to be associated with a sense of déjà entendu (*Mdn* = 0.07, *Range* = 1.00) compared to non-déjà entendu (*Mdn* = 0.00, *Range* = 0.25), *W* = 5019.50, *p* < .001, *r_rb_* = 0.99, *BF_10_* = 4.05 × 10^5^ (note that the Shapiro-Wilk test of normality was significant, *W* = 0.60, *p* < .001).

#### 7.4.8. Commission Errors and Resource Allocation

Following from this logic, the trials accompanied by a “Yes” response that were labeled as being either an instance of a commission error or an omission error were compared to determine whether participants might be using their limited resources to confirm whether their identification attempt was correct. Indeed, the trials on which participants made a commission error were significantly more likely to be associated with a “Yes, use limited resources” response (*Mdn* = 0.33, *Range* = 1.00) compared to the trials on which an omission error was made (*Mdn* = 0.22, *Range* = 0.35), *W* = 3608.5, *p* < .001, *r_rb_* = 0.49, *BF_10_* = 1.84 × 10^3^ (note that we report a Wilcoxon signed-rank test, as the normality assumption was violated based on the Shapiro-Wilk test, *W* = 0.90, *p* < .001).

#### 7.4.9. Commission Errors and Feelings of Curiosity

The patterns of results in Experiment 1 relating to feelings of curiosity for visual stimuli suggested that the trials on which participants make commission errors are also associated with increased curiosity ratings, which may reflect an association between feelings of curiosity and increased internally-directed information-seeking behaviors. To assess whether a similar pattern would emerge in the current experiment using musical stimuli, the curiosity ratings provided on trials associated with a commission error were compared with those associated with an omission error. Indeed, a similar pattern emerged, such that participants provided significantly higher curiosity ratings for test trials associated with commission errors (M = 6.68, SD = 2.62) than test trials associated with omission errors (*M* = 4.50, *SD* = 1.97), *t*(100) = 7.99, *SE* = 0.27, *p* < .001, *d* = 0.80, *BF_10_* = 3.34 × 10^9^ (note that 41 participants were lost from this analysis due to never making a commission error at test).

## 8. General Discussion

In the present study, we present two experiments designed to examine the relationships between metacognitive sensations of closeness to an as yet unretrieved memory (i.e., déjà vu and déjà entendu), curiosity, and subsequent information-seeking behaviors. We specifically examined whether participants experiencing déjà vu-like states would provide higher curiosity ratings and additionally demonstrate altered decision-making behaviors, such as being more inclined to use limited resources to discover the target information or spending longer amounts of time attempting to internally retrieve the target information. In each experiment, we used the RWI paradigm to manipulate whether the current test stimulus, either a novel visual environment or novel song, contained experimentally familiarized features, such as spatial layout (Experiment 1) or tonal sequences (Experiment 2) from the study phase. After each test stimulus was presented, participants were given the opportunity to use limited experimental opportunities to discover the corresponding information from the study phase, even at the risk of being told that there was no corresponding information.

In Experiment 1, we replicated prior findings from the déjà vu literature (e.g., Cleary et al. 2012, 2021a), demonstrating that participants were more likely to report a sense of déjà vu for novel test scenes that contained the same spatial layout as an unrecalled study scene compared to test scenes that did not. In Experiment 2, we demonstrated that participants were more likely to report a sense of déjà entendu for unidentified novel test songs that contained experimentally familiarized tonal information compared to those that did not, extending the prior work examining déjà entendu (e.g., McNeely-White 2020; McNeely-White and Cleary 2019).

Critically, though, the present experiments demonstrate novel findings pertaining to retrieval-failure-based metacognitive states and curiosity as a feeling-of-closeness. When participants reported experiencing déjà vu-like states, which have been considered a metacognitive state reflective of momentary retrieval failure, they concurrently reported heightened levels of curiosity compared to when they did not report experiencing déjà vu-like states. Additionally, instances of déjà vu and déjà entendu were associated with alterations in information-seeking behaviors, both internal and external. While experiencing déjà vu (Experiment 1) or déjà entendu (Experiment 2), participants across both experiments spent longer amounts of time attempting to retrieve the target information, as evidenced by the longer time spent on the Recall prompt. They also made significantly more commission errors, suggesting increased internal search efforts to conjure up potentially relevant target information. Finally, they were also more likely to expend their limited experimental opportunities to potentially receive the target information and thus resolve their metacognitive sensations of curiosity and déjà vu or déjà entendu. Overall, these findings demonstrate that metacognitive sensations of déjà vu-like states are associated with increased curiosity and information-seeking behaviors. Our experimental findings have implications for the mechanisms that may give rise to curiosity as a feeling-of-closeness and also the potential purpose that the metacognitive states of déjà vu and déjà entendu may serve in the larger memory system.

Emerging research within the metacognitive domain has begun to suggest that curiosity may emerge due to metacognitive processes, sometimes emerging as a metacognitive signal to the experiencer that their perceived knowledge gap may be close to resolution (e.g., Brooks et al. 2021; Kang et al. 2009; Litman and Jimerson 2004; Metcalfe et al. 2017, 2020, 2022). In his formal theory proposing that curiosity may arise due to metacognitive monitoring, Litman (2019) proposed that curiosity may emerge when the experiencer perceives a gap in their current knowledge state and the target knowledge state, which in turn motivates them to resolve the gap. From a metacognitive perspective, these two states (perceiving a gap and attempting to resolve the gap) might be viewed as monitoring and control processes. In support of this perspective, research has indeed demonstrated that metacognitive states often associated with momentary retrieval failure but simultaneously feeling close to retrieval success (e.g., TOT) are linked with heightened levels of curiosity. In their 2017 study, Metcalfe et al. demonstrated that the retrieval-failure-based metacognitive sensation of TOT is associated with heightened levels of curiosity and inclinations to spend limited resources to resolve the state. In the present experiments, we provide further support for the viewpoint that curiosity can emerge as a feeling-of-closeness, signaling to the experiencer that they should continue in their search efforts for the target information, either internally or externally, particularly during the metacognitive states of déjà vu and déjà entendu.

From a theoretical perspective, these relationships may be emerging due to the participant detecting that their knowledge gap is small, therefore making it attainable to achieve the desired knowledge state if further search efforts are undertaken (Loewenstein 1994). According to Loewenstein’s information gap theory of curiosity, the size of the perceived knowledge gap is inversely related to the intensity of experienced curiosity, with small perceived gaps in knowledge producing intense levels of curiosity, which in turn motivate information-seeking behaviors. The results found in the present experiments are consistent with Loewenstein’s theory. During instances of déjà vu or déjà entendu, the experiencer is feeling both intense levels of subjective familiarity and novelty, which signals to them that, although there are unknowns about the current situation and they have not yet resolved the gap in knowledge, resolution is possible, as the familiarity sensations signal to the experiencer that there is relevant knowledge held within their memories which can potentially be accessed given further searching. Additionally, based on Litman and Jimerson’s (2004) interest-deprivation theory of curiosity, moments in which participants detect that they do not know something (i.e., curiosity as a feeling-of-deprivation) should be associated with intense levels of curiosity, as the experiencer is detecting that the information will meaningfully increase their knowledge structures and be rewarding to obtain.

In light of the region of proximal learning framework of curiosity, it may be that during feelings of déjà vu or déjà entendu, the participant is within their region of proximal learning and therefore feels highly curious and motivated to resolve the gap (Metcalfe 2023; Metcalfe et al. 2017, 2020, 2022). When presented with the test probe, either a visual scene or a song clip, the participant may sometimes detect a signal that *something* about the current situation is highly familiar. This familiarity-detection in turn may be taken to indicate that relevant information likely resides within memory, making the needed information feel close to conscious access, thereby motivating the person to continue in their search efforts, either internally or externally, to resolve the state. Based on the region of proximal learning framework, participants should experience the strongest levels of curiosity when their metacognitive judgments reflect a state of *almost* knowing the answer (i.e., curiosity as a feeling-of-closeness), and déjà vu-like states may afford a feeling of *almost* knowing something. Along these lines, Cleary et al. (2019) suggested that déjà vu might be akin to feeling on the “tip of an experience,” whereby one feels as if on the verge of recalling how the entire current episode is about to unfold. All in all, the present results are consistent with these ideas, as instances in which participants reported a sense of non-déjà vu or non-déjà entendu were associated with lower levels of curiosity compared to instances in which participants *did* report a sense of déjà vu or déjà entendu, perhaps suggesting that participants felt close to resolving the momentary knowledge gap that was perceived to be small during these strange metacognitive states.

An important future direction is to focus on untangling the temporal dynamics of retrieval-failure-based metacognitive sensations, curiosity as a feeling-of-closeness, and information-seeking behaviors that might in turn lead to successful retrieval. In the current experiments, we were unable to collect the dynamically unfolding mental events occurring as the participant processed a test stimulus. Based on the region of proximal learning framework of curiosity, people should be the most curious when they detect that they *almost* know the answer to a question and therefore engage in search efforts to achieve that knowledge state. In our current experiments, might it be that during the timeframe of the test stimulus unfolding before the participant, they initially experienced retrieval-failure, sensed that something about the test stimulus was highly familiar, which triggered a sense of curiosity, which in turn led to internal search efforts that eventually resulted in successful retrieval? Though the current experimental design does not allow us to address this question (as participants were only prompted at the end of a given trial to indicate their metacognitive sensations, and instances of recall success that may have followed initial metacognitive sensations are undifferentiable from instances of recall success that occurred seemingly instantly upon being presented with a test stimulus), the proposition is plausible and could potentially be examined with an experimental design aimed at capturing temporal dynamics of mental experiences. An example is the Think Aloud approach (e.g., Austin and Delaney 1998). 

The importance of disentangling these sequences can be seen in the present data. In Experiment 1, without considering featural overlap or reported déjà vu state, participants provided the highest curiosity ratings when they successfully recalled the corresponding information from study (*Mdn* = 5.11, *Range* = 10.00) compared to when they failed to recall the corresponding information (*Mdn* = 3.26, *Range* = 9.34), *W* = 1926.00, *p* < .001, *r_rb_* = 0.64, *BF_10_* = 2.28 × 10^3^. These findings, at first glance, are in stark contrast with the current perspectives on curiosity and retrieval status (e.g., Loewenstein 1994; Metcalfe et al. 2020, 2022). According to the region of proximal learning framework of curiosity, instances of correctly knowing the answer should be accompanied by lower levels of curiosity—why would one be curious about something that they already know? Indeed, as shown by Metcalfe et al. (2022), instances of correctly identifying the answer to a general knowledge question were accompanied by lower levels of curiosity compared to instances of failing to identifying the answer, suggesting that participants were curious to discover the unidentified answer. However, upon closer consideration, might this discrepancy be due to the nature of the tasks? In typical experiments examining curiosity, participants are presented with static stimuli, whether it be a general knowledge question (e.g., Kang et al. 2009; Metcalfe et al. 2017, 2020, 2022; Wade and Kidd 2019; Witherby and Carpenter 2022) or a face (Brooks et al. 2021). Once the probe appears, the participant is able to rapidly compile relevant internal information and make a metacognitive decision, which is quickly captured by the experimenters, as the relevant prompts appear soon after the probe. However, in the present study, the potential processes are complicated due to the temporally dynamic nature of the stimuli. When a test probe is presented, such as a virtual environment or a song, the participant is receiving unfolding information as time goes on, perhaps engaging in multiple compilation processes of relevant information from both the dynamically unfolding external probe playing out before them and their internal search efforts. This potential process of receiving dynamically unfolding external information that is incorporated into their compilation of evidence is not captured in the current experiments, as the participant is only prompted at the end of the trial to indicate their metacognitive judgements and retrieval attempts. Might it be that some of these trials associated with identification success were initially instances of retrieval *failure* accompanied by familiarity or a déjà vu-like state, but due to the nature of the temporally dynamic probes and the timing of the experimental prompts, we are unable to capture those search processes? If curiosity does indeed breed information-seeking behaviors during instances in which the participant feels that they *almost* know the answer, then it would be plausible to assume that the patterns of results previously reported (the increased curiosity ratings during instances of retrieval success as opposed to retrieval failure) are largely due to the metacognitive control processes that curiosity engenders—the participant engaged in search efforts that eventually led to retrieval success, perhaps via self-generation of candidate information, about which the participant may have been uncertain as to its correctness (thereby seeking feedback). Future research should focus on disentangling the dynamics of curiosity and search efforts during temporally unfolding stimuli, such as through the aforementioned Think Aloud approach. 

The patterns of results presented in the current study also have implications for the potential adaptive purposes that retrieval-failure-based metacognitive states serve within the larger memory system. In their 2016 chapter, Schwartz and Cleary proposed that one reason people might experience metacognitive sensations during retrieval failure, such as TOT or déjà vu, is due to the memory system detecting that something is amiss. The sensations may serve as a signal to action, indicating to the experiencer that something about the current situation is relevant to stored memories, and with additional metacognitive control efforts, such as internally-directed attention for retrieval attempts or externally-directed attention to one’s environment for relevant cues and clues, the discrepancy might be resolved. Within the TOT literature, such an adaptive association has been demonstrated, with researchers presenting experiments establishing the connections among curiosity and information-seeking behaviors with TOT states (e.g., Litman 2005; Metcalfe et al. 2017). 

Within the déjà vu literature, though, such evidence has not yet been put forth until the present experiments. We demonstrate that déjà vu and déjà entendu are indeed associated with heightened levels of curiosity, which result in participants engaging in additional external and internal search efforts. While experiencing déjà vu-like states, participants across both experiments tended to expend their limited experimental opportunities to resolve the metacognitive sensations and also tended to exhibit increased internally-directed search efforts, such as increased time spent attempting to retrieve the target answer and increased rates of commission errors. These results are the first to formally provide support for the proposal put forth by Schwartz and Cleary (2016), demonstrating that déjà vu-like states may indeed signal to the experiencer to engage in additional search efforts to further complete their memory and knowledge structures for the external world. Future research should continue to examine other ways in which déjà vu-like states are associated with adaptive cognitive functions and memory outcomes.

Future research should also examine how déjà vu-like states might impact subsequent memory as a consequence of the associated curiosity and information-seeking behaviors. For example, might an initial experience of déjà vu or déjà entendu later make an item more memorable or recognizable? That is, if one is indeed engaging in increased internally-directed search efforts while experiencing déjà vu-like states for a given stimulus, might they be further strengthening any existing memory traces (and new memory traces created due to the present encoding experience) for that stimulus, which in turn will be more easily activated upon subsequent presentation of the same stimulus? One possible outcome of experiencing déjà vu-like states and accompanying curiosity and information-seeking behaviors might be that one is better able to recognize that original stimulus that prompted the sense of déjà vu, which may suggest that not only does déjà vu serve as a metacognitive signal to make additional search efforts, but also serves as an initial signal to further enforce one’s memory structures in order to avoid such a knowledge discrepancy again. Prior research within the curiosity domain, such as Kang et al. (2009), has demonstrated that initial curiosity for an item is associated with later memory success.

Beyond the metacognition domain, the present results also have novel implications for the curiosity domain, specifically by providing a mechanism by which curiosity as a feeling-of-closeness might emerge. In his 1950 *relative novelty* proposal, Berlyne suggested that some forms of curiosity might emerge due to the current novel probe containing a subset of familiar features, creating a strange juxtaposition of old and new, signaling to the experiencer that there is an aspect of an old object that they do not yet know. Although Berlyne’s early research demonstrated some support for such a circumstance to prompt feelings of curiosity (e.g., Berlyne 1954, 1958), there has been little research experimentally investigating the proposal since its original development. The present experiments provide direct support for such a circumstance under which curiosity can emerge. Across both experiments, we presented participants with novel test stimuli, such as virtual environments or songs, that potentially contained experimentally familiarized features, such as spatial relations among objects or tonal sequences. Upon encountering these situations, regardless of whether they were able to identify the original source of the familiarity, participants were significantly more curious about these stimuli compared to those that did not contain experimentally familiarized sub-features. Further, in Experiment 2, the magnitude of the relationship between feelings of curiosity and the experimental manipulation of feature exposure (Exposure 0x, Exposure 1x, Exposure 3x) correspondingly increased, such that as the level of featural match between one’s prior memory traces increased, so did their perceived feelings of curiosity, perhaps suggesting that some forms of curiosity emerge due to feature-matching processes. These patterns of results are similar to those found within the literature examining features held within memory traces and the use of familiarity-detection in recognition memory decisions (e.g., Huebert et al. 2022; McNeely-White 2020; McNeely-White et al. 2021, 2022; Ryals and Cleary 2012), demonstrating that as the level of featural overlap between the current probe, whether it be a song or a non-word, and prior memory traces created during the encoding phase increases, the level of familiarity-intensity during retrieval failure correspondingly increases, signaling to the experiencer that, even though they are momentarily experiencing retrieval failure, it is likely that the target information is indeed held within memory, as evidenced by the intense feelings of familiarity. Collectively, the patterns of results found in the present study, in conjunction with those previously reported in the literature, provide support for Berlyne’s *relative novelty* proposal, as they offer a potential mechanism by which perceived familiarity and novelty may prompt a momentary sense of curiosity to resolve the strange sensations. 

In consideration of the present findings, such as the significant, positive correlations between feelings of familiarity and curiosity during retrieval failure, there is now reason to speculate that curiosity as a feeling-of-closeness may be supported by similar mechanisms as those that support familiarity-detection during retrieval failure, namely, feature-matching. Future research should continue to examine the relationships between feature-matching, familiarity-detection, and curiosity as a feeling-of-closeness, both in their shared mechanisms and potential biases. For example, feelings of familiarity during retrieval failure have been shown to bias participants into believing that they can recollect details despite being unable to do so, known as illusory recollection (e.g., Huebert et al. 2022, 2023). Might feelings of curiosity confer a similar bias? The present experiments demonstrated associations between intense feelings of curiosity on trials associated with commission errors as opposed to omission errors. Given the associations between feelings of familiarity and illusory recollection, it may be that curiosity as a feeling-of-closeness might confer an illusory sense of being able to recollect details, as evidenced by the increased rates of commission errors. Future research should examine whether such an association does indeed exist, as it might have implications for participants’ information-seeking behaviors and when they decided to end their search efforts. If participants are highly confident in their “recollected” details, they may potentially decide to conclude their search efforts if not given the feedback that their provided answer was actually incorrect.

Although the present results were primarily interpreted within the context of the region of proximal learning framework (Metcalfe 2023; Metcalfe et al. 2020), an alternative way of interpreting them might be within the conflict detection approach put forward by Gruber and Fandakova (2021). According to this approach, curiosity arises when a conflict is detected between one’s expected outcome and the experienced outcome. This is not unlike a recent approach to déjà vu, whereby it has been argued that déjà vu may arise from the detection of conflict between an experience with a situation and the expectation regarding that situation (Urquhart et al. 2021). That is, perhaps the expectation is that the situation is novel, yet it is experienced as familiar, leading to the detection of a conflict in need of resolution. Perhaps in the present study, this type of conflict detection is at work, leading to the information-seeking behaviors that were observed in the present study. Future research should investigate this possibility.

Finally, the present study may have relevance for the study of consciousness. Neisser et al. (2023) argue that memory *experience* constitutes a critical piece of the puzzle of human consciousness. Déjà vu and déjà entendu represent an experiential aspect of memory, often in the absence of memory content. These phenomena (and the feelings of curiosity that they engender) involve the conscious sense or feeling of a memory, thereby potentially providing a unique window from which to explore the essence of human consciousness. 

## 9. Conclusions

The current experiments investigated the relationships between feelings of curiosity and metacognitive states, specifically déjà vu and déjà entendu. These experiments not only served as a means to further support the proposal that curiosity may be a form of metacognition, but also to inform both curiosity theory concerning the mechanisms underlying curiosity and metacognition theory as to the adaptive function of retrieval-failure-based metacognitive sensations. When presented with a test stimulus, such as a novel visual environment (Experiment 1) or a novel song (Experiment 2), that contained experimentally familiarized features from the study phase, such as spatial relation features or tonal features, participants provided significantly higher curiosity ratings, suggesting that curiosity can emerge due to featural overlap. Additionally, participants provided the highest curiosity ratings while experiencing déjà vu or déjà entendu, which subsequently lead them to be more inclined to use limited resources to uncover relevant information. Overall, the results inform curiosity theory by suggesting a mechanism by which curiosity as a feeling-of-closeness might emerge and also metacognitive theory by suggesting that déjà vu and déjà entendu may be adaptive in that they encourage additional search efforts, both internal and external.

## Figures and Tables

**Table 1:** The proportion of song clips correctly identified, either fully or partially, during the study and test phases.

Exposure Condition	Study		Test			
	*M*	*SD*	*M*	*SD*	*Mdn*	*Range*
0x	-	-	0.02	0.03	0	0.11
1x	0.19	0.12	0.09	0.09	0.07	0.39
3x	0.25	0.13	0.14	0.12	0.12	0.57

## Data Availability

The data presented in this study are publicly available on the Open Science Framework at https://osf.io/9j82m/ (accessed on 29 July 2022).

## Notes

1 Although Metcalfe et al. (2017) allowed their participants in their TOT experiment to view the target answer on 10% of the trials, there is little precedent in the literature concerning how scarce these limited resources must be. Therefore, in the current experiment, the number of limited opportunities to receive relevant information concerning the current test stimulus (20%) was an attempt to create a sense of scarcity so that participants would carefully allocate these opportunities while also allowing a sufficient number of trials to examine metacognitive sensations during these instances.

2 The analyses concerning information-seeking behaviors reported above suggest that participants do indeed use their limited resources strategically, as demonstrated by the increased likelihood that participants reserved their limited resources for trials on which they experienced a sense of déjà vu, felt intense levels of curiosity, and/or were presented with a test scene containing experimentally familiarized spatial features. However, the analyses reported above included *all* trials, regardless of whether the participants had opportunities remaining. Although these analyses provide evidence for significant effects, there may be patterns of results that are hidden due to the inclusion of trials on which no more opportunities remained. For example, a participant may have been experiencing retrieval failure for a given test scene that contained experimentally familiarized spatial features, may have been experiencing déjà vu, high levels of familiarity, and high levels of curiosity; however, if they had already used up their limited resources on prior trials that they would have otherwise used on the current trial, then this would be an inaccurate depiction of their resource allocation behaviors. Therefore, the analyses reported in Supplementary file S3 focus only on trials in which participants still had limited opportunities remaining (i.e., they had one or more opportunities remaining).

3 In Experiment 1, participants were allowed to use their limited opportunities on 20% of the trials, whereas in Experiment 2 they were allowed to do so on 25% of the trials. This discrepancy is largely due to the fact that each test list in Experiment 2 consisted of 12 isolated tonal sequences, which does not divide evenly by 0.20 (that would result in 2.4 opportunities per test list). Additionally, as previously stated, there is little precedent in the literature concerning how scarce these limited resources must be.

4 Note that, due to the programming error described in the Methods section above, 31 of the participants received the incorrect Study Block #5, and therefore the fifth test block was excluded from all analyses for these participants. This exclusion was also taken into account when computing these participants’ resource allocations. Additionally, one participant did not complete the seventh test block, and therefore did not use any of their limited opportunities on those trials.

## Supplementary Materials

The following supporting information can be downloaded at: https://www.mdpi.com/article/10.3390/jintelligence11060112/s1, All supplementary materials, including stimuli, experiment files, and appendices, can be found on the Open Science Framework at https://osf.io/9j82m/ (accessed on 29 July 2022).
