# Supplementary material for: Piquing Curiosity: Déjà vu-Like States Are Associated with Feelings of Curiosity and Information-Seeking Behaviors
Source: J Intell. 2023 Jun 5;11(6):112. doi: 10.3390/jintelligence11060112 (PMC10299614; doi:10.3390/jintelligence11060112)
Supplement: Supplementary file 1 [file jintelligence-11-00112-s001.zip › SupplementaryS1_Experiment1Instructions.pdf]

Supplementary Materials File S1

Below are the exact instructions provided to participants in Experiment 1.

In this experiment, you will see a series of video tours. During the study phase, while watching each video, you will hear a voice tell you what the scene is. For example, while viewing a golf course, the voice would say "This is a golf course. Golf course." Simply watch each scene and try to remember it. When the first set of video tours is over, you will be presented with the test phase.

During the test phase, you will view short virtual tours of scenes again, but this time, they will be new ones not seen in the previous study phase. Some of the virtual tours will resemble scenes from the study phase while others will not. After you view each tour, you will be asked several questions about it. First, you will be asked if the test scene prompted you to feel a sense of déjà vu (the feeling of having been someplace or done something before, without being able to pinpoint why, and despite knowing that the current situation is new). You will indicate Yes or No for this question. Next, you will be asked if the test scene feels familiar to you on a scale of 0 (Not familiar at all) to 10 (Extremely familiar). You will then be asked to rate how curious you are about the scene of a scale of 0 (Not at all curious) to 10 (Extremely curious). Following from this, you will be asked to indicate if you can think of a scene from the study phase that reminds you of the current test scene, and if so, type in the name of that scene. Finally, you will be asked to indicate whether or not you would like to use your limited opportunities to see information concerning the current test scene (Y=Yes, N=No). Specifically, you can request to see the study scene, if any, that corresponds to the current test scene in addition to seeing the name of the scene. However, know that you can only request to receive this information on 20% of the trials.
