# Supplementary material for: Piquing Curiosity: Déjà vu-Like States Are Associated with Feelings of Curiosity and Information-Seeking Behaviors
Source: J Intell. 2023 Jun 5;11(6):112. doi: 10.3390/jintelligence11060112 (PMC10299614; doi:10.3390/jintelligence11060112)
Supplement: Supplementary file 1 [file jintelligence-11-00112-s001.zip › SupplementaryS2_Experiment2Instructions.pdf]

Supplementary Materials File S2

Below are the exact instructions provided to participants in Experiment 2.

In this experiment, you will hear a list of full song clips. This is the study segment of the experiment. As you listen to the songs, you should try your best to identify and remember each of them. When the study segment is over, you will be presented with a memory test.

During the test segment, you will hear the isolated tonal sequences of songs. Some of these isolated tonal sequences will have had their whole, unaltered versions presented at study while others will not have. After each isolated tonal sequence is presented, you will be asked whether you are experiencing déjà entendu, the feeling of having heard something before despite knowing that you have not heard it before (Y=Yes, N=No). Afterwards, you will be asked to judge how familiar the isolated tonal sequence seems to you on a scale of 0 (Not at all familiar) to 10 (Extremely familiar), with the idea being that if you had just heard the whole, unaltered version of this test song clip presented at study, the test song clip will seem more familiar to you. Additionally, you will be asked to rate how curious you feel about the isolated tonal sequence on a scale of 0 (Not at all curious) to 10 (Extremely curious). You will then be asked if you can identify the name of the isolated tonal sequence, if you can. Finally, you will be asked to decide if you would like to use your limited opportunities to receive information concerning the current isolated tonal sequence. Specifically, you can request to hear the whole, unaltered version of the current isolated tonal sequence in addition to seeing the name of the song. However, you can only request to receive this information on 25% of the test trials.
