# Supplementary material for: Piquing Curiosity: Déjà vu-Like States Are Associated with Feelings of Curiosity and Information-Seeking Behaviors
Source: J Intell. 2023 Jun 5;11(6):112. doi: 10.3390/jintelligence11060112 (PMC10299614; doi:10.3390/jintelligence11060112)
Supplement: Supplementary file 1 [file jintelligence-11-00112-s001.zip › SupplementaryS3_Experiment1_LimOppsConditionalized.pdf]

Supplementary Materials File S3

Below we present the analyses conducted on Experiment 1, specifically examining participants' information-seeking behaviors while they still had limited opportunities remaining. Collectively, these results are highly similar to those reported in the main manuscript.

Information-Seeking Behaviors when Opportunities Remained

Expenditure of Limited Opportunities as a Function of Déjà vu Reports

To assess the probability of participants using their limited resources during retrieval failure as a function of déjà vu state, we next report the analysis in which the trials analyzed were those on which participants still had remaining limited opportunities. As found before, participants were significantly more likely to indicate "Yes, use limited resources" during reported déjà vu states (M = .41, SD = .24) than non-déjà vu states (M = .12, SD = .13), $t(67) = 8.96$, SE = .03, $p < .001$, d = 1.09, $BF_{10} = 1.79 \times 10^{10}$. This pattern of results is similar to that reported above in which *all* trials were analyzed, further suggesting that déjà vu may serve as an adaptive signal for participants, as it may motivate them to engage in information-seeking behaviors.

Expenditure of Limited Opportunities as a Function of Spatial Layout

To examine how participants used their limited resources to discover the source scene during instances of retrieval failure as a function of spatial layout, we compared the probabilities of participants responding "Yes, use limited resources" among scenes that shared a spatial layout with an unrecalled scene from study compared to the probabilities of responding "Yes, use limited resources" among scenes that did not share a spatial layout with any scenes from study, specifically while participants still had resources remaining. As was found in the main results, when participants were

presented with a test scene that contained spatially similar features from study, they were not significantly more likely to expend their resources (M = .21, SD = .15) compared to when presented with a test scene that did not contain spatially similar features from study (M = .18, SD = .11), $t(68)$ = 1.71, SE = .01, p = .09, BF_{01} = 1.90. However, note that, like in the results reported in the main manuscript, the means are in the predicted direction and the Bayes Factor only provides anecdotal evidence in favor of the null hypothesis.

We also examined participants' decisions when recall success had occurred, comparing the probabilities of responding "Yes, use limited resources" for test scenes that shared spatial layouts with scenes from study that participants either successfully recalled or failed to recall. When presented with a test scene that did indeed contain spatially similar features from study, participants were significantly more likely to respond "Yes, use limited resources" for scenes to which they successfully recalled the corresponding study scene (Mdn = .32, $Range$ = 1.00) compared to those for which they failed to recall the corresponding study scene (Mdn = .21, $Range$ = .71), W = 582.50, p = .004, r_{rb} = .42, BF_{10} = 30.81 (note that the normality assumption was violated, W = .95, p = .01).

Collectively, these patterns of results are similar to those presented in the main manuscript in which *all* trials were included, suggesting overall that participants tend to use their limited resources to discover information about novel test scenes that contain experimentally familiarized spatial features, and that they tend to use their resources in order to receive confirmatory feedback, as demonstrated by their increased use of resources on trials in which identification succeeded.

Relationship between Expenditure of Limited Opportunities and Curiosity Ratings

The relationship between subjective feelings of curiosity and participants' decisions to use limited resources was demonstrated in the main manuscript when analyzing *all* trials, such that participants provided significantly higher curiosity ratings on trials for which they decided to use their limited resources compared to when they did not. To assess whether the magnitude of this relationship would increase when only focusing on trials in which participants still had opportunities remaining, a Wilcoxon signed-rank test was conducted (note that the normality assumption was violated, $W = .94$, $p = .003$), comparing participants' average curiosity ratings for trials on which they did versus did not decide to use their limited resources. Indeed, participants provided significantly higher curiosity ratings for trials on which they decided to use their resources (*Mdn* = 5.18, *Range* = 10.00) compared to when they decided against using their resources (*Mdn* = 2.65, *Range* = 9.29), $W = 2227.00$, $p < .001$, $r_{rb} = .96$, $BF_{10} = 2.27 \times 10^6$.
