# Supplementary material for: Piquing Curiosity: Déjà vu-Like States Are Associated with Feelings of Curiosity and Information-Seeking Behaviors
Source: J Intell. 2023 Jun 5;11(6):112. doi: 10.3390/jintelligence11060112 (PMC10299614; doi:10.3390/jintelligence11060112)
Supplement: Supplementary file 1 [file jintelligence-11-00112-s001.zip › SupplementaryS4_Experiment2_IDStatusConditionalizedAnalyses.pdf]

Supplementary Materials File S4

As discussed in the manuscript, the identification rates found in the present study were notably lower than those found by Kostic and Cleary (2009). Given that their study was conducted over a decade before the present study, the differences in baseline identifiability of the unaltered musical pieces in the study phase could indicate generational differences among the participants' pre-existing knowledge of the particular melodies used across the two studies (with younger generations possibly being less familiar with these particular musical pieces). In order to ensure that if participants had been able to recall a particular studied melody in response to the test cue, they would be able to indicate so by naming it, Kostic and Cleary had conditionalized their test trials on identifiability at study (with the logic being that if participants were thinking of the song itself in their heads, they would then be able to name it because they were able to do so in response to the unaltered song at study). However, because fewer unaltered study phase songs were identified in the present study, we did not have as many trials in which to do this across our conditions. Because of this, along with the fact that Kostic and Cleary found that the conditionalized data followed the same pattern as the unconditionalized data in their study, our primary analyses reported in the main Results section of the manuscript (unless otherwise stated) focus on instances of retrieval failure at test regardless of whether participants were able to successfully identify the song at study (unconditionalized).

Following from Kostic and Cleary (2009), below we present the analyses conducted for Experiment 2, in which we conditionalize based on identification status at study *and* test. Specifically, to examine the patterns when the unidentifiable test song clips would have been identifiable if not reduced to an isolated set of features, we examined trials for which the identification status at *study* was either full or partial song identification success while identification status at *test* was song identification failure. Note that the reported analyses

below necessarily excluded many participants, as there were few instances in which identification of the unaltered song clip at study succeeded then later failed at test for the song's isolated feature set. Also, it was rare for participants to successfully identify a song clip at study and then make a commission error at test, resulting in the majority of trials being lost. Therefore, we do not report these analyses below due to insufficient statistical power to make meaningful conclusions.

Déjà Entendu Reports

While experiencing retrieval failure for an isolated tonal sequence that originally had its whole song clip identified at study, participants reported experiencing a sense of déjà entendu on 55% (*SD* = .31) of the trials.

Probability of Déjà Entendu given Test Song Clip Feature Familiarization

A one-way repeated-measures ANOVA examining the influence of Exposure Condition (Exposure 0x, Exposure 1x, Exposure 3x) on the probability of reporting déjà entendu for an unidentified isolated tonal sequence at test that had been identified in its whole version at study (for those that *did* correspond to a song presented at study), a significant effect emerged, $F(1.68, 175.16) = 21.99$, $MSE = .09$, $p < .001$, $n_p^2 = .18$, $BF_{10} = 6.84 \times 10^6$. Note that Mauchly's test of sphericity was significant, with an ε of .84, $\chi^2(2) = 23.55$, $p < .001$. We therefore applied the Huynh-Feldt corrections. Unidentified isolated tonal test sequences that corresponded to an identified whole, unaltered song clip presented once at study (Exposure 1x) led to a greater likelihood of reporting a sense of déjà entendu ($M = .55$, $SD = .36$) compared to unidentified isolated tonal sequences that did not correspond to any whole, unaltered song from the study phase (Exposure 0x; $M = .39$, $SD = .20$), $t(104) = 4.08$, $SE = .03$, $p < .001$, $d = .47$, $BF_{10} = 2.82 \times 10^3$. However, there was no significant difference in the probability of reporting a sense of déjà entendu for an unidentified isolated tonal test sequence that corresponded to an identified whole,

unaltered study song clip presented once (*Mdn* = .50, *Range* = 1.00) during the study phase compared to three separate times at study (Exposure 3x; *Mdn* = .67, *Range* = 1.00), *W* = 1325.00, $p = .08$, $BF_{01} = 1.11$ Note, though, that the results are in the predicted direction and the Bayes Factor does not provide conclusive evidence in favor of one hypothesis over the other.

Familiarity Ratings as a Function of Test Song Clip Feature Familiarization

A one-way repeated-measures ANOVA examining the influence of Exposure Condition (Exposure 0x, Exposure 1x, Exposure 3x) on subjective familiarity ratings provided to unidentified isolated tonal test sequences that potentially mapped onto an identified whole, unaltered study song was conducted. A significant effect was found, $F(1.90, 197.39) = 39.92$, $MSE = 3.21$, $p < .001$, $n_p^2 = .28$, $BF_{10} = 3.24 \times 10^{12}$. Note that the Huynh-Feldt correction was applied, as we found Mauchly's test of sphericity to be significant, $\chi^2(2) = 7.73$, $p = .02$, with an ε of .95. When participants heard an unidentified isolated tonal test sequence that corresponded to a previously identified whole, unaltered song presented during the study phase (Exposure 1x; *Mdn* = 5.00, *Range* = 10.00), they provided significantly higher familiarity ratings compared to when they heard an unidentified isolated tonal test sequence that did not correspond to any song presented during the study phase (Exposure 0x; *Mdn* = 3.69, *Range* = 6.85), *W* = 1144.00, $p < .001$, $r_{rb} = .57$, $BF_{10} = 1.32 \times 10^4$ (note that the normality assumption was violated, *W* = .98, $p = .04$). Similarly, participants provided significantly higher familiarity ratings for unidentified isolated tonal test sequences that corresponded to an identified whole, unaltered song presented three separate times throughout the study phase (Exposure 3x; *Mdn* = 5.67, *Range* = 10.00) compared to only once, *W* = 1508.00, $p < .001$, $r_{rb} = .40$, $BF_{10} = 21.75$.

Test Song Clip Curiosity Ratings as a Function of Déjà Entendu Reports

When presented with an unidentified isolated tonal test sequence that potentially mapped onto an identified whole, unaltered song clip presented at study, participants provided significantly higher curiosity ratings while concurrently experiencing déjà entendu ($M = 5.66$, $SD = 1.89$) compared to when not ($M = 2.98$, $SD = 2.12$), $t(138) = 17.08$, $SE = .16$, $p < .001$, $d = 1.45$.

Curiosity Ratings as a Function of Song Clip Feature Familiarization

A one-way repeated-measures ANOVA performed on curiosity ratings given to unidentified isolated tonal test sequences that potentially mapped onto an identified whole, unaltered song presented at study revealed a significant effect, $F(1.76, 182.74) = 9.79$, $MSE = 3.75$, $p < .001$, $n_p^2 = .09$, $BF_{10} = 200.44$. Huynh-Feldt corrections were applied, as the sphericity assumption was not met, with an ε of .88, $\chi^2(2) = 17.44$, $p < .001$. Curiosity ratings provided to unidentified isolated tonal sequences that did not correspond to a whole, unaltered song presented at study (Exposure 0x; $Mdn = 4.04$, $Range = 9.68$) were significantly lower than those provided to unidentified isolated tonal sequences that corresponded to an identified whole, unaltered song presented at study (Exposure 1x; $Mdn = 5.00$, $Range = 10.00$), $W = 1771.50$, $p = .01$, $r_{rb} = -.31$, $BF_{10} = 7.88$. However, only a marginally significant increase was found when comparing the curiosity ratings provided to unidentified isolated tonal sequences that corresponded to an identified whole, unaltered song presented at study three separate times (Exposure 3x; $Mdn = 5.00$, $Range = 10.00$) compared to those that only corresponded to an identified whole, unaltered song presented once at study, $W = 1822.50$, $p = .065$, $r_{rb} = .22$, $BF_{01} = 1.63$ (although note that the results are in the predicted direction and the Bayes Factor only provides anecdotal evidence in favor of the null hypothesis).

The Relationship between Familiarity Ratings and Feelings of Curiosity

A significant, positive correlation was found between participants' curiosity (*Mdn* = 4.11, *Range* = 9.54) and familiarity ratings (*Mdn* = 3.62, *Range* = 7.04) provided to unidentified isolated tonal test sequences that potentially mapped onto an identified whole, unaltered song presented at study. A one-sample Wilcoxon signed-rank test using 0 as the critical value showed that the two measures were significantly and positively correlated, with an average correlation of .60 (*SD* = .33, *Mdn* = .69, *Range* = 1.55), *V* = 9403, $p < .001$, $r_{rb} = .96$, $BF_{10} = 8.17 \times 10^7$. Shapiro-Wilk's test for normality was significant, *W* = .86, $p < .001$.

Limited Resources as a Function of Déjà Entendu Reports.

When presented with an unidentified isolated tonal test sequence that potentially mapped onto an identified whole, unaltered song presented at study, participants were significantly more likely to want to use their limited resources on trials associated with déjà entendu (*Mdn* = .33, *Range* = 1.00) compared to non-déjà entendu (*Mdn* = .05, *Range* = .38), *W* = 8496.50, $p < .001$, $r_{rb} = .94$, $BF_{10} = 5.80 \times 10^6$. Note that the normality assumption was violated, *W* = .98, *p* = .01.

Resource Allocation as a Function of the Song Clip Feature Familiarization.

A one-way repeated-measures ANOVA using the Huynh-Feldt correction (ε = .88; Mauchly's test: $\chi^2(2) = 5.98$, *p* = .05) examined the influence of feature familiarization condition (Exposure 0x, Exposure 1x, Exposure 3x) on the probability of using resources to discover information about the unidentified isolated tonal sequence that potentially mapped onto an identified whole, unaltered song presented at study. This analysis revealed a significant effect, $F(1.93, 200.44) = 7.20$, *MSE* = .06, $p < .001$, $\eta_p^2 = .07$, BF_{10} = 28.77. Participants were significantly more likely to indicate "Yes, use limited resources"

for unidentified isolated tonal sequences that corresponded to an identified whole, unaltered song presented once during the study phase (Exposure 1x; *Mdn* = .20, *Range* = 1.00) compared to unidentified isolated tonal sequences that did not correspond to any whole, unaltered study song (Exposure 0x; *Mdn* = .15, *Range* = .33), W = 1818.50, p = .045, r_{rb} = .24, BF_{10} = 1.73, although the Bayes Factor only provides anecdotal evidence in favor of the alternative hypothesis (note that the normality assumption was violated, W = .88, p < .001). However, no significant difference was found between the probability of wanting to expend limited resources in the Exposure 1x condition and the Exposure 3x condition (Exposure 3x; *Mdn* = .20, *Range* = 1.00), W = 1411.00, p = .79, BF_{01} = 8.71 (note that the normality assumption was violated, W = .96, p = .01).

As was found in Experiment 1, participants were again more likely to indicate "Yes, use resources" on trials associated with identification success (*Mdn* = .27, *Range* = 1.00) compared to identification failure (*Mdn* = .18, *Range* = .38), W = 6532.50, p < .001, r_{rb} = .44, BF_{10} = 723.73 (note that the normality assumption was violated, W = .97, p = .001).

Resource Allocation and Curiosity Ratings

When presented with an unidentified isolated tonal sequence that potentially mapped onto an identified whole, unaltered song presented at study, participants provided significantly higher curiosity ratings when they also decided to use their limited resources (M = 6.28, SD = 2.14) compared to when they decided against using their resources (M = 3.60, SD = 2.09), $t(133)$ = 14.20, SE = .19, p < .001, d = 1.23, BF_{10} = 1.70 x 10^{25}.

Resource Allocation and Familiarity Ratings

Overall, when participants were presented with an unidentified isolated tonal sequence that potentially mapped onto an identified whole, unaltered song presented at study, they provided significantly higher familiarity ratings on trials when they decided

to use their limited resources (M = 5.95, SD = 2.24) compared to when they decided to not use their limited resources (M = 3.18, SD = 1.56), $t(133) = 15.11$, SE = .18, $p < .001$, d = 1.31, $BF_{10} = 2.66 \times 10^{27}$.

Time Spent at Retrieval Prompt

When examining the reaction time data on the Recall prompt, specifically on trials associated with omission errors, a significant difference was found in the amount of time spent as a function of reported déjà entendu state. Specifically, when participants were attempting to identify an isolated tonal test sequence that potentially corresponded to an identified whole, unaltered song presented at study, but ultimately made an omission error on the Recall prompt, they spent a significantly longer time on the Recall prompt when in a reported déjà entendu state (Mdn = 1347.67 ms, $Range$ = 6873.69 ms) compared to when they were in a reported non-déjà entendu state (Mdn = 1236.89 ms, $Range$ = 4956.03 ms), W = 6021.00, p = .01, r_{rb} = .26, BF_{10} = 3.73 (note that the normality assumption was violated, W = .80, $p < .001$).
